# Social network strategies to distribute HIV self‐testing kits: a global systematic review and network meta‐analysis

**DOI:** 10.1002/jia2.26342

**Published:** 2024-07-24

**Authors:** Siyue Hu, Fengshi Jing, Chengxin Fan, Yifan Dai, Yewei Xie, Yi Zhou, Hang Lv, Xi He, Dan Wu, Joseph D. Tucker, Weiming Tang

**Affiliations:** ^1^ Dermatology Hospital of Southern Medical University Guangzhou China; ^2^ School of Public Health Southern Medical University Guangzhou China; ^3^ University of North Carolina Project – China Guangzhou China; ^4^ Faculty of Data Science City University of Macau Taipa China; ^5^ School of Public Health Nanjing Medical University Nanjing China; ^6^ Programme in Health Services and Systems Research, Duke‐NUS Medical School Singapore; ^7^ Zhuhai Center for Diseases Control and Prevention Zhuhai China; ^8^ Zhuhai Xutong Voluntary Services Center Zhuhai China; ^9^ London School of Hygiene and Tropical Medicine London UK

**Keywords:** HIV, HIV self‐testing, social network, intervention, network meta‐analysis, systematic review

## Abstract

**Introduction:**

Social network strategies, in which social networks are utilized to influence individuals or communities, are increasingly being used to deliver human immunodeficiency virus (HIV) interventions to key populations. We summarized and critically assessed existing research on the effectiveness of social network strategies in promoting HIV self‐testing (HIVST).

**Methods:**

Using search terms related to social network interventions and HIVST, we searched five databases for trials published between 1st January 2010 and 30th June 2023. Outcomes included uptake of HIV testing, HIV prevalence and linkage to antiretroviral therapy (ART) or HIV care. We used network meta‐analysis to assess the uptake of HIV testing through social network strategies compared with control methods. A pairwise meta‐analysis of studies with a comparison arm that reported outcomes was performed to assess relative risks (RR) and their corresponding 95% confidence intervals (CI).

**Results:**

Among the 4496 manuscripts identified, 39 studies fulfilled the inclusion criteria, including one quasi‐experimental study, 22 randomized controlled trials and 16 observational studies. Networks HIVST testing was organized by peers (distributed to known peers, 15 studies), partners (distributed to their sexual partners, 16 studies) and peer educators (distributed to unknown peers, 8 studies). Among social networks, simulating the possibilities of ranking position, peer distribution had the highest uptake of HIV testing (84% probability), followed by partner distribution (80% probability) and peer educator distribution (74% probability). Pairwise meta‐analysis showed that peer distribution (RR 2.29, 95% CI 1.54−3.39, 5 studies) and partner distribution (RR 1.76, 95% CI 1.50−2.07, 10 studies) also increased the probability of detecting HIV reactivity during testing within the key population when compared to the control.

**Discussion:**

All of the three social network distribution strategies enhanced the uptake of HIV testing compared to standard facility‐based testing. Linkage to ART or HIV care remained comparable to facility‐based testing across the three HIVST distribution strategies.

**Conclusions:**

Network‐based HIVST distribution is considered effective in augmenting HIV testing rates and reaching marginalized populations compared to facility‐based testing. These strategies can be integrated with the existing HIV care services, to fill the testing gap among key populations globally.

**PROSPERO Number:**

CRD42022361782

## 1 INTRODUCTION

Human immunodeficiency virus (HIV) testing is considered as a significant stage of the HIV care continuum and prevention services [1]. Knowledge of HIV status contributes to timely treatment and prevents wider transmission of HIV. Globally, approximately 5.46 million people living with HIV are still unaware of their HIV status [2]. This gap is not solely limited by healthcare system constraints but is also influenced by factors such as HIV knowledge, fear and stigmatization, posing significant obstacles to meet global goals [3, 4]. Efficient and effective approaches to HIV testing services are needed to expand HIV testing. As a complementary approach to HIV testing services (HTS), HIV self‐testing (HIVST) has demonstrated its safety, accuracy and acceptability among key populations [5, 6]. The significance of HIVST is underscored in the latest consolidated guidelines on HIV, while the WHO has further emphasized recommending network‐based strategies in their updated recommendations [7, 8]. Thus, strategies that can facilitate the effectiveness of HIVST are needed to achieve the maximum impact of HIV testing.

Social network strategy, a method or approach used in social interventions that operates through interconnections among key populations, has great potential to improve HIVST coverage [9]. Social network‐based HIV testing approaches are an extension of HIV partner services. Trained providers recruit people living with HIV or those who are HIV negative and at ongoing risk of HIV as “seeds” through which they encourage and invite individuals in their sexual, drug injecting or social networks to participate in voluntary HTS [7, 10]. Social network testing approaches have been recommended by CDC, WHO and other guidelines, and are increasingly being used as part of a comprehensive package of care and prevention [8, 11].

There is a strong theoretical underpinning for why social networks are helpful in health behaviour programming. Social network theory examines the relationships and interactions between social groups, communities and their various networks. Centrality refers to how densely an individual is connected to others in his or her network [12]. In the social network context, peers share the same characteristics (e.g. demographic, cultural, health outcomes and behaviours) with the target audience [13]. Consequently, members of social networks often have similar HIV acquisition risk, trust each other and are interested in helping each other [14]. Based on these principles, our social network strategies utilize the egocentricity and drive of the initial seed in which seeds (with or without prior knowledge) take on the role of HIV prevention educators, HIVST kit providers or health partners to help identify network partners and motivate others to get tested. Information can be disseminated more effectively when health information for preventive measures are disseminated through close social relationships [15]. Using close friends or trusted peers as a source of information increases the credibility and trustworthiness of the information for the recipients [16]. In addition, social network strategies utilize social contacts and reciprocal relationships to encourage individuals to adopt healthy behaviours and provide support when needed [17, 18]. Therefore, it is essential to carefully consider the selection of distributors, distribution methods and recipients of HIVST when implementing social networking strategies.

Previous studies have mainly evaluated the efficiency of HIVST, which have shown higher test uptake rates compared to standard testing services alone [6]. Meanwhile, studies that have explored the role of social networks have generally only compared HIV testing or risk behaviours and have not further explored the impact of social networks on HIVST [17, 19]. Some of these studies have been limited to marginalized populations such as female sex workers [20]. Currently, conversations around HIV testing are also focused on integrating HIVST as a strategy within the continuum of HIV care, and social network strategies have shown great potential in improving HIVST [18, 21], while there is an expansion of network‐supported HIVST. However, there is a lack of reviews that summarize the role of social network strategies in HIVST interventions. Knowledge gaps remain in terms of which strategies are most effective, for whom and in which settings they are best suited for scaling up. To optimize the effectiveness of HIVST, it is essential to consider information about the different role of distributors and distribution methods within the social network in the HIV care cascade. Network meta‐analysis allows for simultaneous comparison of the effects of multiple interventions and consideration of other potential sources of heterogeneity.

This study aims to investigate the effects of social network strategy on improving HIV testing uptake among key populations who used HIVST through a systematic review and network meta‐analysis.

## 2 METHODS

### 2.1 Search strategy and selection criteria

This systematic review and meta‐analysis followed the Preferred Reporting Items for Systematic Reviews and Meta‐Analyses for Network Meta‐Analyses (PRISMA‐NMA) and Cochrane guidelines [22]. The following databases were utilized for the literature search: (1) PubMed; (2) Embase; (3) Web of Science; (4) Cochrane Library; and (5) Wiley. The initial search strategy formulated for PubMed used the combination of key terms that include “HIV or AIDS,” “social network” and “HIVST” (Appendix S1), which were subsequently adapted into corresponding index terms for the other searched databases. We included all studies that involved social networks to distribute HIVST kits, where there was a common relationship or behavioural link was used for distribution. The search was completed and limited to peer‐reviewed journal articles published in English from 1st January 2010 to 30th June 2023, with no geographic limitation. Additional articles were identified through manual reference checking of relevant studies.

Trials with a comparison arm or observational studies that evaluated any social network strategies used for HIVST in any setting that reported quantitative outcomes were included. The study population was the population receiving HIVST services. All studies were required to use social network strategies as an intervention and to report outcomes on HIV testing uptake, HIV prevalence rates, or linkage to antiretroviral therapy (ART) or HIV care. ART initiation was selected preferentially as an outcome, and linkage to any HIV services was used if ART initiation was not available. Studies without a comparison arm were also included. Two independent reviewers (YD and CF) first assessed the title and abstracts to identify relevant records for inclusion following the eligibility criteria, with a third reviewer as a tiebreaker (SH). Full texts of included studies were retrieved and assessed for inclusion following the same screening method. Two reviewers jointly developed a data extraction form and completed it independently.

### 2.2 Intervention categorization

Social network‐based HIV testing approaches are an extension of HIV partner services. In our social network‐based HIV testing, participants (seeds, defined as “index”) will be recruited and encouraged to distribute HIVST kits to members who are linked by a common set of relationships or behaviours (defined as “alter”), regardless of the HIV status of the index. We grouped social network strategies for HIVST according to who distributed the tests, yielding three distribution strategies: (1) peer distribution focused on recruiting peers and encouraging peers to distribute the HIVST kits to people in their social networks (known peers); (2) partner distribution that distributed the kits from participants to their sexual partners; and (3) peer educator distribution that peer educators distribute the services to people assigned to their group (unknown peers) (Table 1). As a platform for providing HIVST kits, social networks for peer and partner distribution provide a channel for communication and diffusion. Peer educators were usually assigned to recruit participants or were randomly assigned to groups of recruited participants after receiving corresponding training, and then linkages were established for behavioural interventions. The biggest difference between peer educators and peers for our definition was whether there was a basis for pre‐existing contact. Peers spontaneously distribute kits within their social networks based on pre‐existing contacts, whereas peer educators were primarily randomly assigned to groups of recruited participants and then established connections for behavioural interventions. Peer educators acted as popularizers of HIV‐related information and providers of HIVST kits, further distributing HIVST kits by influencing social norms in the established communities.

**Table 1:** HIVST distribution strategy in social network strategies

HIVST distribution strategy	Index	Alter	Connection
**Peer distribution**	Peer	People in their social networks (known peers)	Pre‐existing social contact
**Partner distribution**	Participants	Sexual partners	Pre‐existing sexual contact
**Peer educator distribution**	Peer educators	People assigned to their group (unknown peers)	Post‐established contact

### 2.3 Data analysis

Seeds distributed HIVST kits to members of their social networks, with a sample size that was the sum of the number of seeds and the number of social network members. For studies with a comparison arm, pooled relative risks (RR) with 95% confidence intervals (CI) were used to compare outcomes, and the heterogeneity was assessed by calculating I^2^. We built forest plots for each outcome using Review Manager version 5.4. A network meta‐analysis was performed to analyse the primary outcome of HIV testing uptake. The final model was selected by evaluating a combination of the deviance information criterion, Markov chain Monte Carlo error, and trace and density plots. The node‐splitting model was applied to analyse the direct and indirect comparison results to observe the consistency. Results were presented in risk ratios (RR) and 95% credible intervals (CrI), as well as relative effects tables and forest plots. In addition, direct comparisons were used to generate indirect effect estimates and ranking distribution strategies provided a complementary approach to determining optimal implementation strategies. Ranking probabilities were used to indicate the likelihood or chance of each intervention being ranked at a specific position within the comparison. A ranking probability of 1 (100%) represents the highest ranking of a distribution strategy and 0 the lowest. All network meta‐analyses were carried out using the meta and gemtc packages in R software version 4.2.2.

### 2.4 Quality assessment

In an analysis of quality assessment, studies were stratified based on study design and level of evidence. Bias among randomized controlled studies were assessed across five domains using the Cochrane risk of bias tool [23]. Bias in observational studies was assessed using the Newcastle‐Ottawa Quality Assessment Scale [24]. The quality of observational studies was evenly distributed, with some studies judged to be of poor or fair quality, mainly due to insufficient comparability and outcome data (Table S5). For all randomized controlled trials (RCTs), performance and detection bias items represented a high risk of bias, respectively, primarily due to failure to blind participants and personnel and to blind outcome assessment (Table S6 and Figure S1). A quasi‐experimental study identified concerns about selection and reporting bias due to selective reporting.

## 3 RESULTS

**Figure 1:**
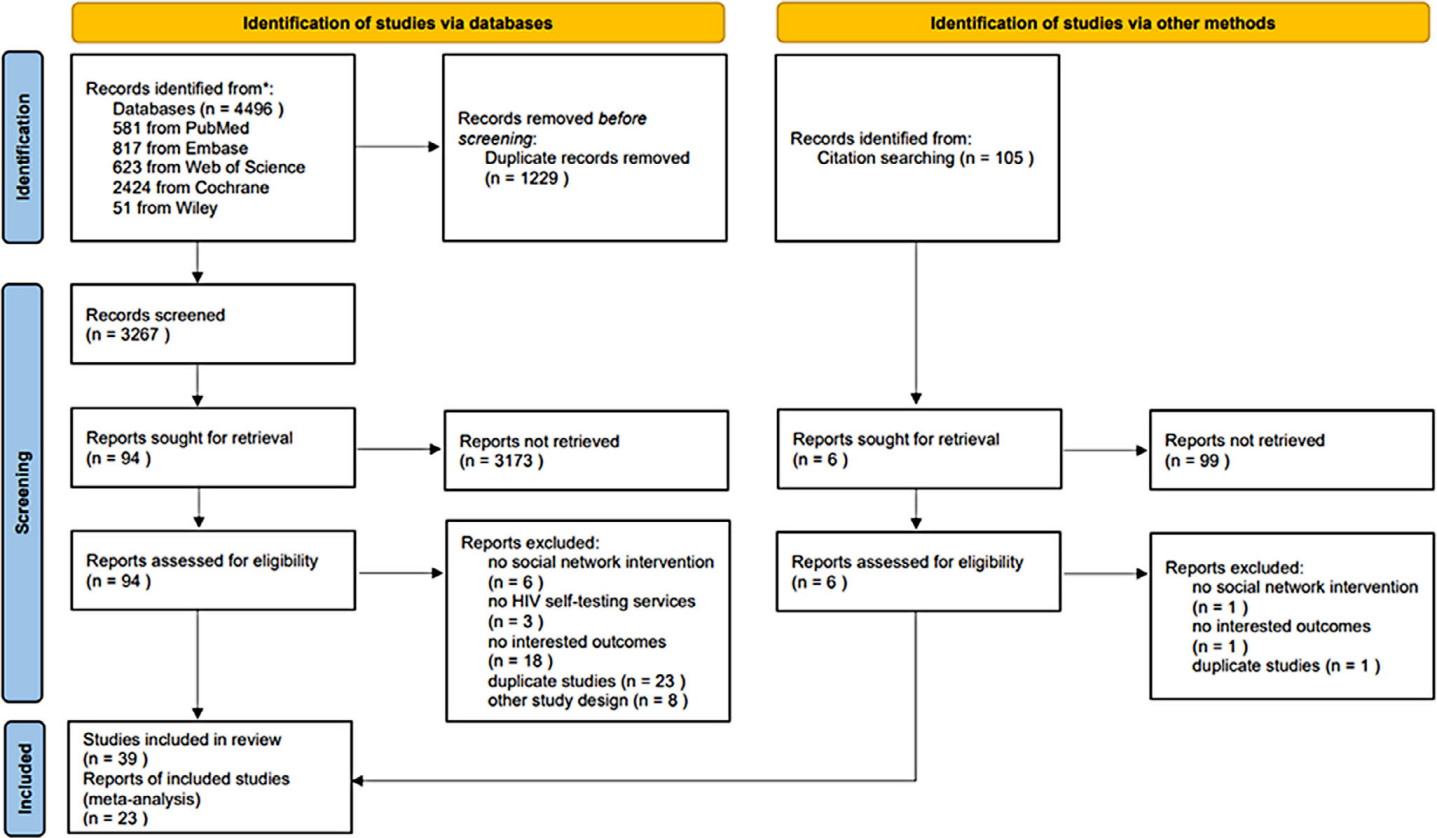
PRISMA‐NMA flow chart for study.

The search yielded 4496 citations and 105 additional records identified through other sources. After removing duplicate citations, 3267 unique titles and abstracts were screened, and 100 full‐text articles were assessed for eligibility. This evaluation ultimately included 39 studies, including one quasi‐experimental study, 22 RCTs and 16 observational studies (Figure 1). Six studies were from high‐income countries, 24 in middle‐income countries and nine in low‐income countries. The included studies were conducted in eight countries from 2010 to 2021, seven in China [25, 26, 27, 28, 29, 30, 31], six in South Africa [32, 33, 34, 35, 36, 37], six in the United States [38, 39, 40, 41, 42, 43], six in Uganda [44, 45, 46, 47, 48, 49], eight in Kenya [50, 51, 52, 53, 54, 55, 56, 57], three in Malawi [58, 59, 60], two in Vietnam [61, 62] and one in Zambia [63].

The study populations were: 16 conducted in men who have sex with men (MSM), seven in male partners of antenatal care clinic (ANC), two in key populations and their partners (including people who inject drugs, MSM, sex workers and their partners), two in female sex workers, two in young people, three in young woman and their partners, two in women living with HIV and their male partners, one in partners of people living with HIV, one in HTC general population and three in two populations. Among them, two were conducted in partners of people living with HIV and male partners in ANC, and one in both key populations and sexual partners of pregnant and lactating women.

Of the 39 included studies, 26 had comparison groups, of which 22 studies compared HIVST distribution with standard healthcare worker (HCW)‐administered facility‐based HIV testing, one study compared different forms of the secondary distribution of HIVST and three studies compared the impact of peer educators on HIVST distribution. Regarding compensation, 22 studies had no compensation, whereas 12 studies paid their partners and five studies imposed specific requirements. Of 12 studies paid their partners, 11 giving monetary incentives and one giving material incentives, including reimbursement for transportation, T‐shirts, backpacks and umbrellas. In addition, the five specific requirements were that two had to advise on partner negotiation and communication, one required telephone confirmation that the kit had been delivered to the recipient, one required a report for confirmatory HIV testing and counselling (HTC) at the clinic and one required daily or on‐demand pre‐exposure prophylaxis (PrEP). The characteristics of the included studies and interventions with and without comparison groups are shown in Tables 2 and 3. Further detailed characterization and study outcomes are listed in the Supplementary Material (Tables S1 and S2).

**Table 2:** Included study characteristics: no comparison group

Study	Country	Study design	Study population	HIVST distribution strategy	Sample size^a^
Kitenge 2022	South Africa	Cross‐sectional study	General population	Peer distribution	1089
Kwan 2023	China	Cross‐sectional study	MSM	Peer distribution	463
Lippman 2018	South Africa	Cohort	MSM	Peer distribution	855
Li 2021	China	Cross‐sectional study	MSM	Peer distribution	2263
Matovu 2020	Uganda	Cohort	Young people (both males and females, age 15–24 years) and adult men (25 years or older)	Peer distribution	332
Nasuuna 2022	Uganda	Cross‐sectional study	Key populations (FSW and MSM), sexual partners of pregnant and lactating women (mothers)	Peer distribution/partner distribution	18,756
Nguyen 2019	Vietnam	Cohort	Key populations and their partners (people who inject drugs, MSM, sex workers and their partners)	Peer educator distribution	2009
Nguyen 2019	Vietnam	Cohort	Key populations and their partners (people who inject drugs, MSM, sex workers and partners of people living with HIV)	Peer educator distribution	892
Pintye 2019	Kenya	Cross‐sectional study	Male partners of HIV‐uninfected women	Partner distribution	705
Thirumurthy 2016	Kenya	Cohort	HIV‐uninfected women aged 18–39 years and their male partners	Partner distribution	725
Wu 2021	China	Cohort	MSM	Peer distribution	652
Zhang 2021	China	Cohort	MSM who are PrEP users	Partner distribution	651
Zishiri 2022	South Africa	Cross‐sectional study	Partners of ANC and partners of newly diagnosed HIV	Partner distribution	ANC: 7710 index: 2961

Abbreviations: ANC, antenatal care clinic; FSW, female sex worker; HIV, human immunodeficiency virus; HIVST, HIV self‐testing; MSM, men who have sex with men; PrEP, pre‐exposure prophylaxis.

^a^ Sample size is the sum of seed size and social network member size.

**Table 3:** Included study characteristics: comparison group

Study	Country	Study design	Study population	HIVST distribution strategy	Sample size^a^
Chanda 2017	Zambia	Cluster RCT	FSW	Peer educator distribution	1125
Choko 2019	Malawi	Cluster RCT	Male partners of ANC	Partner distribution	4698
Choko 2021	Malawi	Cluster RCT	Partners of ANC, partners of newly diagnosed HIV	Partner distribution	8505
Dovel 2019	Malawi	RCT	Partners of people living with HIV	Partner distribution	849
Frye 2021	The United States	RCT	Young Black MSM and transwomen	Peer educator distribution	376
Gichangi 2018	Kenya	RCT	Male partners of ANC	Partner distribution	2543
Joseph 2022	South Africa	RCT	Male partners of women living with HIV	Partner distribution	352
Korte 2020	Uganda	Cluster RCT	Male partners of ANC	Partner distribution	2948
Lightfoot 2018	The United States	Cohort	MSM	Peer distribution	195
MacGowan 2020	The United States	RCT	MSM	Peer distribution	2968
Marwa 2019	Kenya	RCT	Male partners of ANC	Partner distribution	1567
Masters 2016	Kenya	RCT	Male partners of ANC	Partner distribution	1140
Merchant 2018	The United States	RCT	Young adult Black, Hispanic and White MSM	Peer distribution	241
Mujugira 2022	Uganda	RCT	Male partners of pregnant women living with HIV	Partner distribution	723
Okoboi 2020	Uganda	Cross‐sectional study	MSM	Peer distribution	165
Ortblad 2017	Uganda	Cluster RCT	FSW	Peer educator distribution	960
Pettifor 2020	South Africa	RCT	Young women and their peers and partners	Peer distribution	1394
Sha 2022	China	Quasi‐experimental study	Gay, bisexual and other MSM	Peer distribution	359
Shahmanesh 2021	South Africa	Cluster RCT	Young women (18–24) and all young people (aged 18–30)	Peer distribution	4220
Thirumurthy 2021	Kenya	Cluster RCT	Male partners of HIV‐uninfected women	Partner distribution	4180
Wango 2023	Kenya	RCT	Male partners of adolescent girls (15–19)	Partner distribution	648
Young 2013	The United States	Cluster RCT	MSM	Peer educator distribution	128
Young 2022	The United States	RCT	MSM	Peer educator distribution	979
Van Der Elst 2017	Kenya	Cohort	MSM	Peer distribution	1038
Zhang 2020	China	RCT	MSM	Partner distribution	636
Zhou 2022	China	RCT	MSM	Peer distribution	653

Abbreviations: ANC, antenatal care clinic; FSW, female sex worker; HCW, healthcare worker; HIV, human immunodeficiency virus; HIVST, HIV self‐testing; MSM, men who have sex with men; RCT, randomized controlled trial; SOC, standard of care.

^a^ Sample size is the sum of seed size and social network member size.

### 3.1 HIVST distribution strategy

We classified the social network distribution strategies into three categories based on the people who distributed the test: peers, partners and peer educators. Overall, 16 studies distributed HIVST kits directly from participants to their sexual partners [28, 31, 34, 37, 45, 49, 50, 51, 53, 54, 55, 56, 57, 58, 59, 60]. As for social network strategies, 15 studies recruited index participants and encouraged participants to distribute HIVST kits to people in their social networks (alters) [25, 26, 27, 29, 30, 33, 35, 36, 38, 42, 43, 46, 47, 48, 52]. Most studies had multiple HIVST kits provided directly to index participants by HCWs for distribution to alters. Two studies used formative research by conducting focus group discussions to collect the necessary data to inform the design of peer‐led HIVST interventions [42, 47]. Peer educators were recruited in eight studies to provide additional interventions [32, 39–41, 44, 61–63]. In most studies, HIVST kits were distributed person‐to‐person, with participants applying for HIVST kits and then distributing them face‐to‐face in their social networks [25, 28, 33–37, 45, 50, 51, 53, 58–60]. In contrast, a subset of studies were based on social media, an HIVST application link on a phone app [26, 27, 29] or a web‐based platform [30, 38, 43] that provides HIVST kits in the form of online ordering and mailing.

### 3.2 Uptake of HIV testing

**Figure 2:**
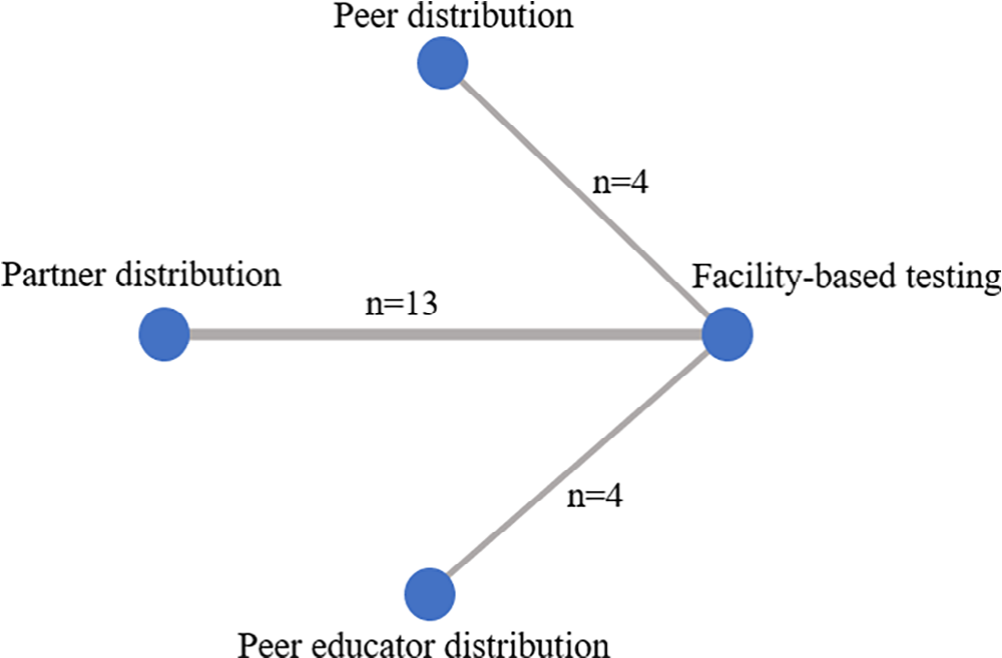
Network map: uptake of HIV testing. Network map represents the number of studies contributing to the direct comparisons in the network.

**Figure 3:**
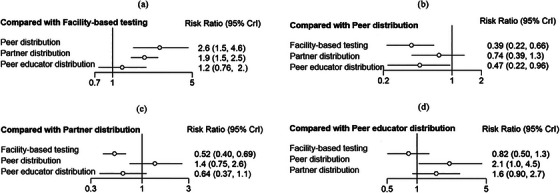
Network estimates of HIV testing uptake.

**Figure 4:**
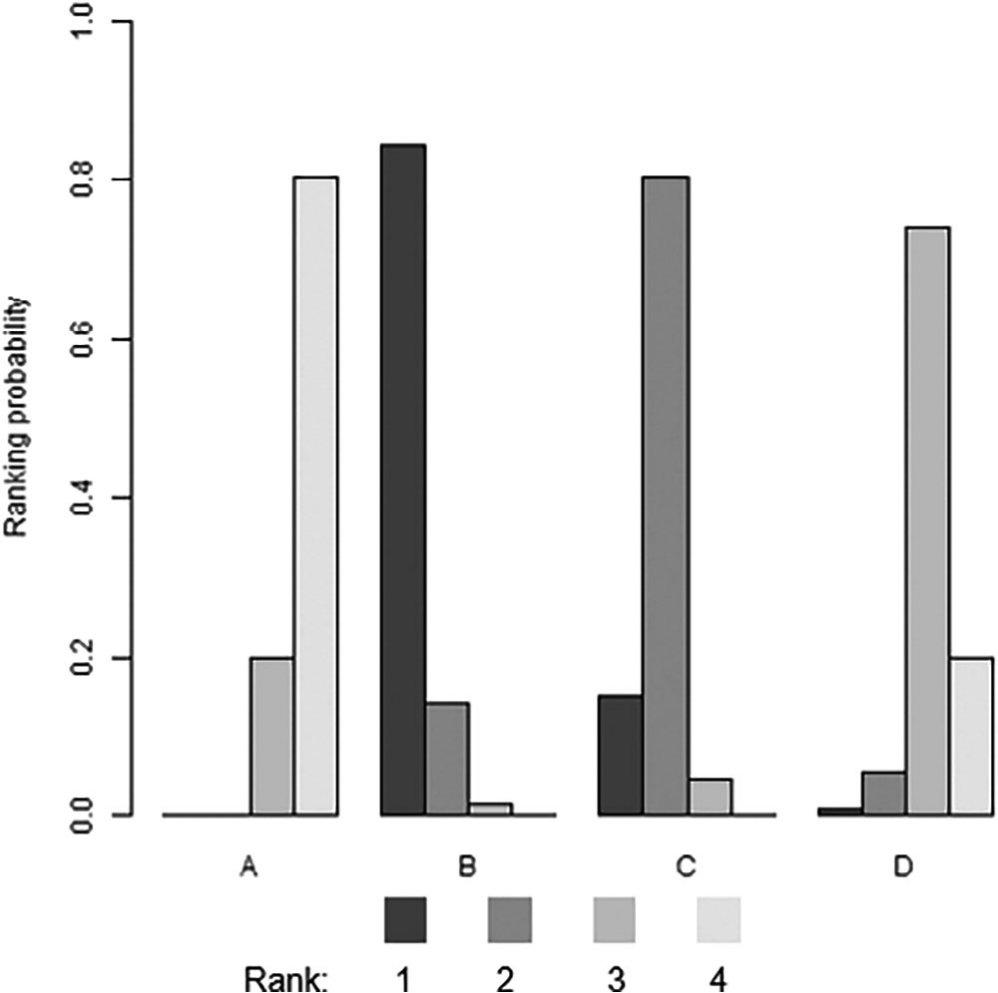
HIV testing strategies ranking probabilities for HIV testing uptake. For each strategy, the coloured bar represents the probability of that strategy being ranked at each position (first, second, third, fourth) among all strategies. Darker colours represent high ranking (most effective); light colours represent low ranking (least effective). (A) Facility‐based testing, (B) Peer distribution, (C) Partner distribution, (D) Peer educator distribution.

Data on the uptake of HIV testing were obtained from 21 studies. Network meta‐analysis directly compared differences in the uptake of HIV testing in HIVST kits distributed by peers (4 studies), partners (13 studies) and peer educators (4 studies) in their social networks with the standard HCW‐administered facility‐based HTS (Figure 2). Network estimates showed that the distribution of peer in social network (RR 2.60, 95% CrI: 1.50−4.60) and partner in sexual network (RR 1.90, 95% CrI: 1.50−2.50) resulted in higher HIV testing uptake than facility‐based testing (Figure 3). However, the effect of peer educator distribution on the increased HIV testing uptake was insignificant (RR 1.20, 95% CrI: 0.76−2.00). Compared with facility‐based (80% of simulations with the last rank), ranking probabilities (Figure 4) showed that HIVST kits had the highest uptake among social networks via peer distribution (84% of simulations with the highest rank), partner distribution (80% of simulations with the second highest rank) and peer educator distribution (74% of simulations with the third highest rank) (Table S3).

**Figure 5:**
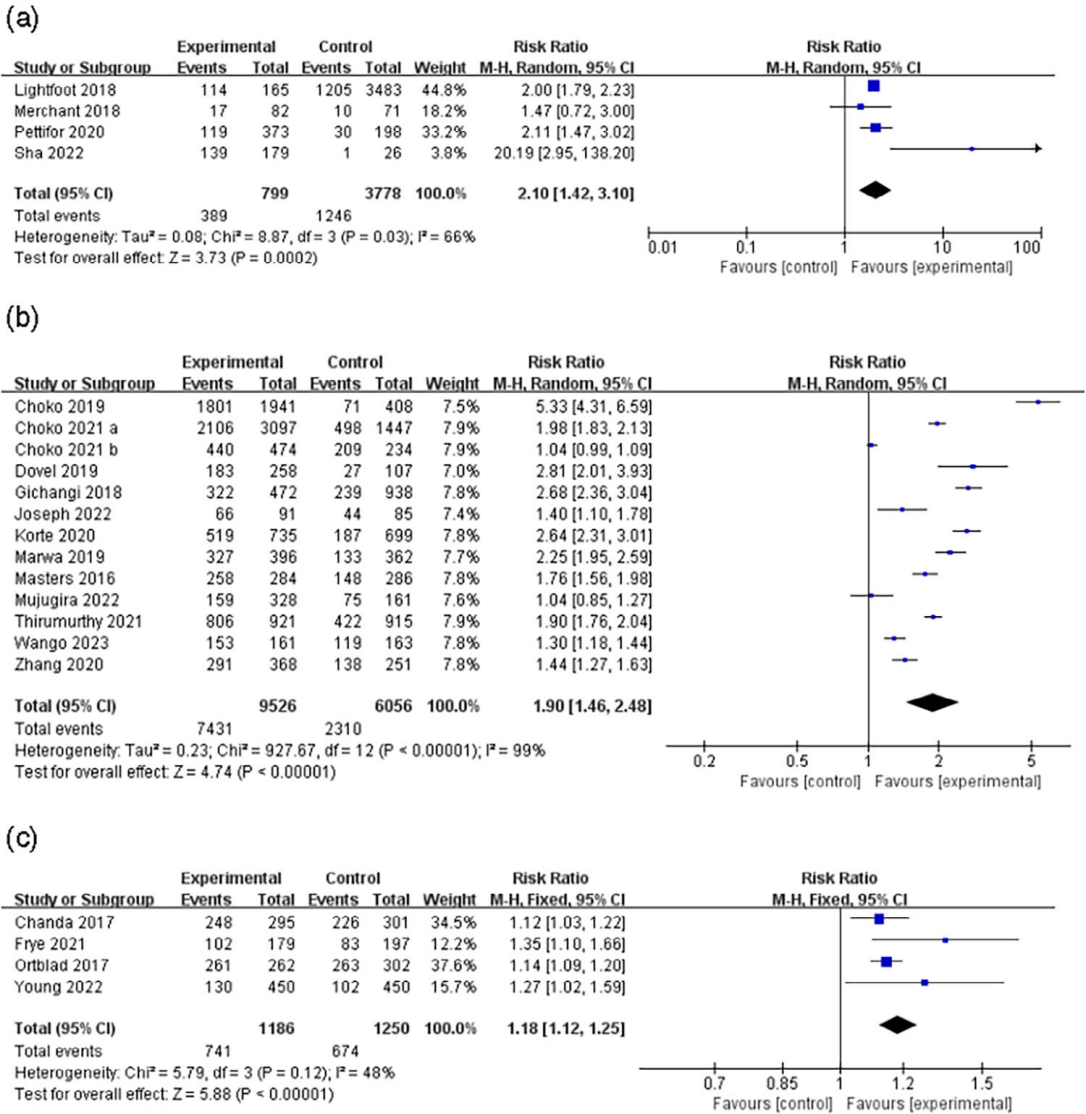
Uptake of HIV testing by distribution strategy. (A) Peer distribution, (B) Partner distribution, (C) Peer educator distribution.

Data from pairwise meta‐analyses also supported this view. Four studies reported uptake of HIV testing through peer distribution [25, 33, 38, 42]. The meta‐analysis showed a doubling of HIV testing uptake compared with standard of care (SOC), the standard facility‐based testing (RR 2.10, 95% CI 1.42−3.10, I^2^ 66%). Data from 12 RCTs showed that HIV testing rates were significantly higher when social network interventions were delivered through sexual partners than in the SOC (RR 1.90, 95% CI 1.46−2.48, I^2^ 99%) [31, 34, 45, 49–51, 55–60]. Similarly, findings showed that the intervention group that used social influence with the help of peer educators to disseminate acquisition risk information and attempted to change social norms with the help of peer educators (RR 1.18, 95% CI 1.12, 1.25, I^2^ 48%) had higher uptake of HIV testing than the comparison group (Figure 5) [39, 41, 44, 63].

### 3.3 HIV prevalence

**Figure 6:**
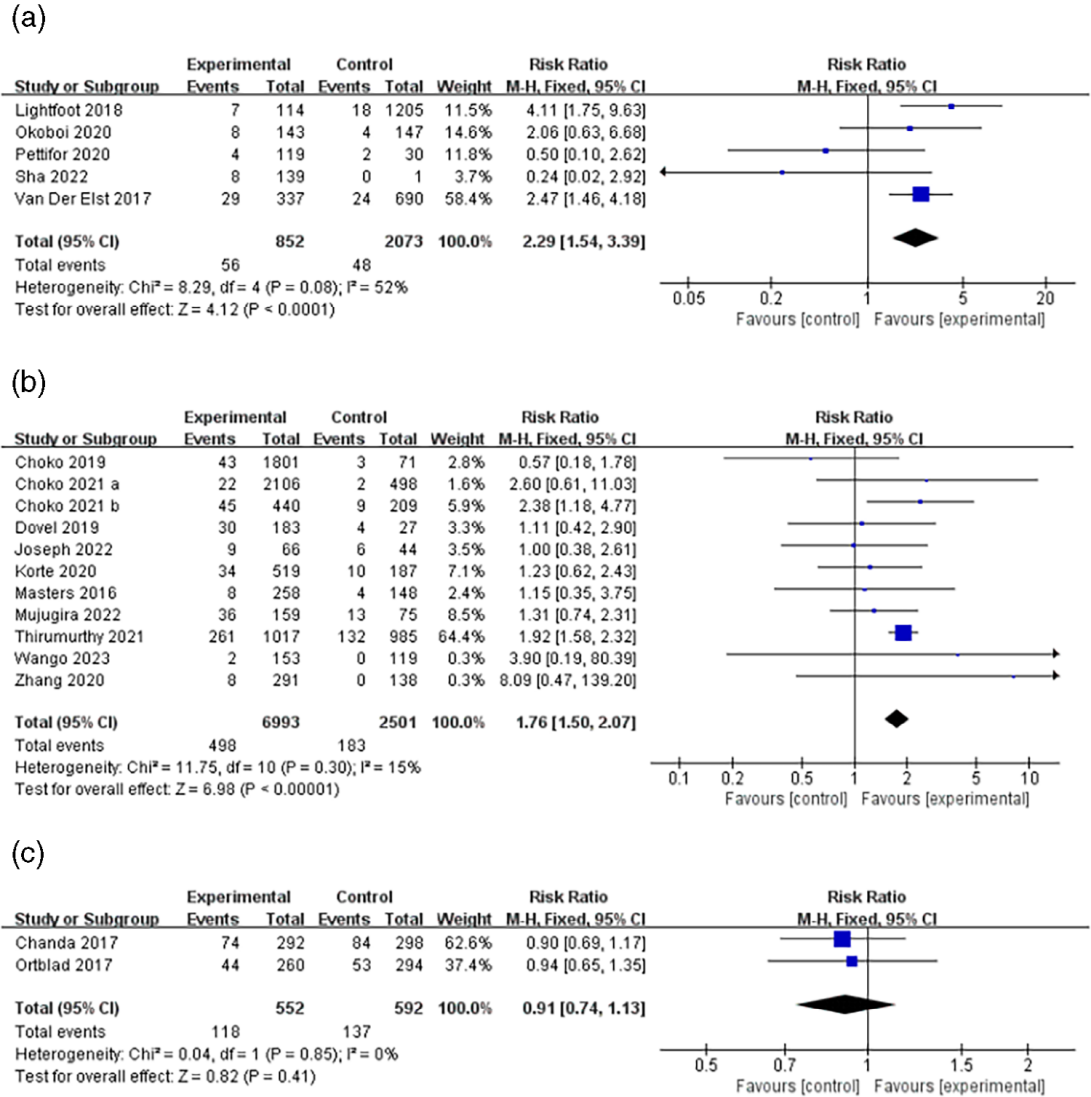
HIV prevalence by HIVST distribution strategy. (A) Peer distribution, (B) Partner distribution, (C) Peer educator distribution.

Overall, 17 studies reported HIV prevalence after HIV testing by comparing HIV reaction rates among alters in social networks who received HIVST kits. Five studies that reported differences in HIV prevalence outcomes based on peer distribution were included [25, 33, 42, 46, 52]. Meta‐analysis showed significantly higher HIV reaction rates among alters using peer distribution compared to SOC (RR 2.29, 95% CI 1. 54−3.39, I^2^ 52%). Data on HIV reaction rates for partner distribution were obtained from 10 studies [31, 34, 45, 49, 51, 55, 56, 58–60]. Interventions using partner‐distributed HIVST kits showed a higher likelihood of detecting HIV reactivity in the partners of participants (RR 1.76, 95% CI 1. 50−2.07, I^2^ 15%). However, when comparing the HIVST distribution strategy using peer educator influence to the comparison group, there appeared to be no difference in the association of finding people living with HIV (RR 0.91, 95% CI 0.74−1.13, I^2^ 0%) (Figure 6) [44, 63].

### 3.4 Linkage to ART or HIV care among people living with HIV

Ten studies reported the linkage to ART or HIV care among people living with HIV found by testing, with five of these studies having more than 70% of people living with HIV initiating ART or linking to HIV care (Table S2) [45, 52, 58, 60, 63]. Four direct comparisons contributed to the meta‐analysis of the impact of distribution strategies on linkage to ART or HIV care among people living with HIV (Table S4). All distribution strategies, whether peer distribution, partner distribution or peer educator distribution, have comparable linkage to care as facility‐based testing (combined three distributions: RR 0.88, 95% CI 0. 75−1.03, I^2^ 2%). A cohort study directly demonstrated the impact of peer distribution strategies for delivering HIVST kits on linkage: although comparable to the impact of facility‐based testing, men who received confirmatory testing after receiving HIVST tended to receive immediate treatment with ART [52].

## 4 DISCUSSION

Our systematic review and meta‐analysis examined HIVST distribution strategies using social network strategies. This study extends the existing literature by using network meta‐analysis to summarize the social network strategies for HIVST, pooling the effectiveness of augmenting testing rates among key populations, and including linkage to care data. Our results indicated that peer and partner distribution strategies effectively increased HIV testing uptake and testing yield compared to traditional HIV testing performed by HCWs in health facilities.

Network‐based HIVST distribution could draw on key population assets and community strengths. The results of our study suggest that social network strategies can effectively promote HIV and increase the uptake of HIV testing among key populations. Consistent with past research, HIVST surmounted multifaceted structural impediments besetting HIV testing services, while social network strategies further enhanced coverage on this basis [6, 21, 64]. Social network strategies focus first on risks, recruiting the first group of HIV acquisition risk or people living with HIV for testing, training and education [17]. Furthermore, social network strategies not only reach these potentially marginalized key populations hidden from current HIV testing practices but also encourage conversations within the social networks about risk behaviours and HIV testing‐related information. Based on this asset‐based framework, the recruited initial peers are considered sources of wisdom, insight and strength. Social networks provide greater access to a wide range of risks, health information and practices, and information can be passed easily and frequently between individuals [65, 66]. In addition, members of the same social network often have similar norms, attitudes and risk behaviours, and social networks can influence risk and health behaviours through a variety of psychosocial mechanisms and linkage characteristics, such as frequency of contact, duration of contact, social influence, social norms and social support [67]. Social networks can be used to promote HIVST and to follow up with self‐testers. The social influence of peer educators can also be used to disseminate HIV acquisition risk information and attempt to change social norms to increase HIV prevention and testing behaviours [39, 40, 41]. Consequently, the systematic integration of social network strategies and leverage of community strength for promulgating HIVST warrants earnest endorsement to attain comprehensive and recurrent HIV testing.

Network meta‐analysis integrated both direct and indirect evidence, providing a more comprehensive understanding of intervention effects. Ranking probabilities indicated the priority of peer distribution in enhancing HIV testing uptake. Partner services represent a crucial component of broader social network strategies for promoting HIVST, leveraging existing sexual partnerships to distribute HIVST kits and promote testing uptake [15, 18]. Our study demonstrated a significant increase in HIV testing uptake through partner distribution, emphasizing the effectiveness of using intimate relationships to expand HIV test coverage. Social network strategies, on the other hand, encompass a wider array of interventions that leverage interconnected social groups or communities beyond sexual partnerships. These strategies utilize existing social ties, such as friendships, peer networks and community affiliations, to disseminate HIVST kits and promote testing uptake among key populations [21]. While partner services focus specifically on leveraging sexual relationships, social network strategies target a broader spectrum of social connections to maximize testing reach and impact.

Our network meta‐analysis showed that HIV testing uptake was higher for distributing HIVST kits through peers and partners than peer educators. Based on pre‐existing contacts, peers and partners spontaneously distribute kits within their social networks [17, 35]. In contrast, peer educators were primarily randomly assigned to groups of recruited participants and then established connections for behavioural interventions [40, 44]. This fact suggests that the distribution strategy of peer educators should be further optimized by considering the dynamics of relationships within the social network of the priority population, combining economic costs and social support [68]. Future refinements of HIVST distribution strategies should account for factors such as motivation, skill proficiency, self‐efficacy, social norms, behavioural patterns and supplementary interventions to augment the effectiveness of the peer educator approach [69].

This systematic review and meta‐analysis showed that the social network intervention was associated with increased testing yield. Consistent evidence suggests that social network intervention has proven effective in identifying undiagnosed HIV‐positive individuals in networks [70, 71]. Compared to HCWs, peers and partners can have more interpersonal interactions with members of their social networks and provide them with more authentic empathy, validation and practical advice, thus providing effective social support for undiagnosed HIV‐positive individuals and facilitating case identification [68]. Knowledge of HIV status contributes to the control and prevention of HIV. Increasing the testing rate of finding people living with HIV is essential to facilitate linkages to ART or HIV care and prevention services for key populations. In addition to assisted partner notification services, case identification should be facilitated by expanding service options, especially through social network strategies to convey relevant information [72].

We also found that social network‐based HIVST distribution strategies and facility‐based testing are comparable in linkage to care. However, social network strategies showed significant improvements in the uptake of testing, which positively impacted linkage. In addition, peer‐based interventions, including communication links, social support and monetary incentives, increased the likelihood of linkage to care [73, 74]. The comparable linkages demonstrated the reliability and potential of the social network strategy in improving HIV care services, not only in HIV testing. Future network studies should further consider and evaluate additional robust interventions to support the linkage between post‐HIVST testing and HIV care.

This systematic evaluation and meta‐analysis have several limitations. First, 37 of the 39 studies had a high or unclear risk of bias in at least one of the methodological quality assessments. Due to the nature of the intervention and social network strategies to distribute HIVST kits, it was difficult to conduct blinded comparisons of participants assigned to study groups or study allocators. Second, the methods used in the comparison group were not entirely consistent. For example, in the comparison group, either no intervention was provided, or HIVST services were promoted without peer educators or the use of social network strategies to provide general health‐related information. We further categorized intervention types to mitigate the heterogeneity and scrutinized measurement heterogeneity before combining the data. Third, the included studies varied widely in terms of intervention duration and time to outcome assessment, leading to potential bias in this meta‐analysis. Finally, the contextual variation between study settings, including sociocultural, economic backgrounds and HIV programmes, may introduce variability, thus affecting the generalizability of our findings. These variations may influence the effectiveness of social network strategies in different regions. Therefore, caution should be exercised when extrapolating results to settings with significantly different contextual factors. Further research considering and exploring these contextual variations is warranted to enhance the understanding of the applicability and adaptability of social network strategies for HIVST in different settings.

The discerned findings strongly and compellingly advocate for the progressive amplification of peer distribution strategies, underpinned by empirical evidence that underscores their remarkable efficacy. First, the assets and community power of the key population should be fully utilized and mobilized. Similarly, in its new recommendations on HIV testing, WHO is calling on countries to increase testing coverage by promoting testing through sexual and social networks [8]. Second, more efficient distribution strategies for peer educators should be further explored, considering economic costs and incorporating the dynamics of relationships within the social networks of the priority population. Future HIV prevention interventions could be carried out in partnership with the community to identify trusted and knowledgeable peer educators and train them to be most effective [75]. Finally, along with increasing testing for early detection of people living with HIV, emphasis should be placed on increasing access to ART or HIV care among key populations. Peers play an important role in providing support for linkage to care services. Different additional interventions, such as home‐initiated ART care [76], conditional monetary incentives [58, 60] and peer educator navigating [32], could be chosen to improve the linkage after HIVST. It is paramount to acknowledge that the potency of social network interventions is contingent on their continual evolution and alignment with evolving paradigms. To harness the true potential of social networks in curbing HIV transmission, it is incumbent to propel dedicated research endeavours and maintain an ongoing regimen of scrutiny and adaptation. These imperative measures are indispensable in not only curbing the onward trajectory of HIV transmission but also in charting a course that fortifies linkage to ART or HIV care subsequent to HIV acquisition.

## 5 CONCLUSIONS

This comprehensive systematic review and meta‐analysis stand as a testament to social network strategies’ viability, acceptance and efficacy in promoting HIVST. The findings therein substantiate the resounding impact of diverse HIVST distribution strategies, universally augmenting the uptake of HIV testing, facilitating the early identification of cases and effectively linking to HIV care. It is necessary to capitalize on the assets and community strengths of the key population. The ascendancy of interventions at the social network stratum extends beyond mere testing proficiency; they furnish a dynamic platform for dispensing services and instigating shifts in social norms. Consequently, these transformations precipitate salubrious changes in risk and health behaviours that orchestrate a positive ripple effect on HIV outcomes.

## COMPETING INTERESTS

The authors declare that they have no conflict of interest.

## AUTHORS’ CONTRIBUTIONS

WT and JDT designed the review. CF, YD and SH identified relevant studies and extracted data. YX, YZ and HL analysed and interpreted data. All other authors (XH, DW, JDT and WT) contributed to data interpretation. SH and FJ wrote the first draft of the article. All authors critically revised the article and approved the final version.

## FUNDING

This work was supported by the Key Technologies Research and Development Program (2022YFC2304900‐4 to WT), National Institute of Health (R34MH119963, 1UG1HD113156‐01, 1R25AI170379‐01 and R01AI158826 to WT), National Nature Science Foundation of China (81903371 to WT) and CRDF Global (G‐202104‐67775 to WT). The funders had no role in study design, data collection and analysis, decision to publish or preparation of the manuscript.

## Supporting information

Supporting Information file 1: Appendix S1. Search terms.

**Table S1(a)**: HIVST distribution strategy details – No comparison group
**Table S1(b)**: HIVST distribution strategy details – Comparison group
**Table S2**: Outcomes of the included studies
**Table S3(a)**: Network meta‐analysis relative effects (league) table of HIVST distribution strategies – HIV testing uptake
**Table S3(b)**: Network meta‐analysis ranking probabilities of HIVST distribution strategies – HIV testing uptake
**Table S4**: Linkage to ART or Any Care Among people living with HIV by Distribution Strategy, Study Design and Population Subgroup
**Table S5**: Risk of bias for included studies – Observational studies
**Table S6**: Risk of bias for included studies–RCTs and a quasi‐experimental study

## Data Availability

The data that support the findings of this systematic review and meta‐analysis are available from the corresponding author upon reasonable request. Please contact Dr. Weiming Tang at weiming_tang@med.unc.edu for access to the data.
