# Supplementary material for: Social network strategies to distribute HIV self‐testing kits: a global systematic review and network meta‐analysis
Source: J Int AIDS Soc. 2024 Jul 24;27(7):e26342. doi: 10.1002/jia2.26342 (PMC11269052; doi:10.1002/jia2.26342)
Supplement: Supplementary file 1 — Supporting Information file 1: Appendix S1. Search terms. [file JIA2-27-e26342-s001.docx]

**Appendix 1**

**Table S1 Search terms**

| **Pubmed (Yield: 581)** | | |
| --- | --- | --- |
| No. | Terms | Comments |
| 1 | (((((((((((((((((((((((((((((((((((((((((((((("HIV")) OR "Acquired Immunodeficiency Syndrome") OR (HIV)) OR (Human Immunodeficiency Virus)) OR (Immunodeficiency Virus, Human)) OR (Immunodeficiency Viruses, Human)) OR (Virus, Human Immunodeficiency)) OR (Viruses, Human Immunodeficiency)) OR (Human Immunodeficiency Viruses)) OR (Human T Cell Lymphotropic Virus Type III)) OR (Human T-Cell Leukemia Virus Type III)) OR (LAV-HTLV-III)) OR (Lymphadenopathy-Associated Virus)) OR (Lymphadenopathy Associated Virus)) OR (Lymphadenopathy-Associated Viruses)) OR (Virus, Lymphadenopathy-Associated)) OR (Viruses, Lymphadenopathy-Associated)) OR (Human T Lymphotropic Virus Type III)) OR (Human T-Lymphotropic Virus Type III)) OR (AIDS Virus)) OR (AIDS Viruses)) OR (Virus, AIDS)) OR (Viruses, AIDS)) OR (Acquired Immune Deficiency Syndrome Virus)) OR (Acquired Immunodeficiency Syndrome Virus)) OR (HTLV-III)) OR (HIV-1)) OR (HIV-2)) OR (HIV1)) OR (HIV2)) OR (Acquired Immunodeficiency Syndrome)) OR (Immunologic Deficiency Syndrome, Acquired)) OR (Acquired Immune Deficiency Syndrome)) OR (Acquired Immuno-Deficiency Syndrome)) OR (Acquired Immuno Deficiency Syndrome)) OR (Acquired Immuno-Deficiency Syndromes)) OR (Immuno-Deficiency Syndrome, Acquired)) OR (Immuno-Deficiency Syndromes, Acquired)) OR (Syndrome, Acquired Immuno-Deficiency)) OR (Syndromes, Acquired Immuno-Deficiency)) OR (Immunodeficiency Syndrome, Acquired)) OR (Acquired Immunodeficiency Syndromes)) OR (Immunodeficiency Syndromes, Acquired)) OR (Syndrome, Acquired Immunodeficiency)) OR (Syndromes, Acquired Immunodeficiency)) OR (AIDS) | HIV/AIDS |
| 2 | "Social Networking" OR "Social Network Analysis" OR "Social Networking" OR "Networking, Social" OR "Social networks" OR "network social" OR "Social network" OR "Social Network Analysis" OR "Analyses, Social Network" OR "Analysis, Social Network" OR "Network Analysis, Social" OR "Social Network Analyses" OR "peer" OR "peer influence" OR "peer leader" OR "peer educator" OR "peer mentor" OR "seed" OR "ego" OR "index" OR "index participant" OR "alter" OR "support" OR "partner" OR "Sexual Partners" OR "partner services" | Social network |
| 3 | "hiv self-testing" OR "hiv self-test" OR "HIVST" OR "home test" OR "rapid test" OR "home self test" OR "Self-test" OR "Self-testing" OR "home test" OR "home testing" | HIVST |
| 4 | (#1 AND #2 AN D #3) AND (with publication date from Jan 2010 to June 2023) |  |
| **Embase (Yield: 817)** | | |
| No. | Terms | Comments |
| 1 | (hiv:ab,ti OR 'human immunodeficiency virus':ab,ti OR 'immunodeficiency virus human':ab,ti OR 'immunodeficiency viruses human':ab,ti OR 'virus human immunodeficiency':ab,ti OR 'viruses human immunodeficiency':ab,ti OR 'human immunodeficiency viruses':ab,ti OR 'human t cell lymphotropic virus type iii':ab,ti OR 'human t cell leukemia virus type iii':ab,ti OR 'lav-htlv-iii':ab,ti OR 'lymphadenopathy-associated virus':ab,ti OR 'lymphadenopathy associated virus':ab,ti OR 'lymphadenopathy-associated viruses':ab,ti OR 'virus, lymphadenopathy-associated':ab,ti OR 'viruses, lymphadenopathy-associated':ab,ti OR 'human t lymphotropic virus type iii':ab,ti OR 'aids virus':ab,ti OR 'aids viruses':ab,ti OR 'virus, aids':ab,ti OR 'viruses, aids':ab,ti OR 'acquired immune deficiency syndrome virus':ab,ti OR 'acquired immunodeficiency syndrome virus':ab,ti OR 'htlv-iii':ab,ti OR 'hiv-1':ab,ti OR 'hiv-2':ab,ti OR 'hiv1':ab,ti OR 'hiv2':ab,ti OR 'acquired immunodeficiency syndrome':ab,ti OR 'immunologic deficiency syndrome, acquired':ab,ti OR 'acquired immune deficiency syndrome':ab,ti OR 'acquired immuno-deficiency syndrome':ab,ti OR 'acquired immuno deficiency syndrome':ab,ti OR 'acquired immuno-deficiency syndromes':ab,ti OR 'immuno-deficiency syndrome, acquired':ab,ti OR 'immuno-deficiency syndromes, acquired':ab,ti OR 'syndrome, acquired immuno-deficiency':ab,ti OR 'syndromes, acquired immuno-deficiency':ab,ti OR 'immunodeficiency syndrome, acquired':ab,ti OR 'acquired immunodeficiency syndromes':ab,ti OR 'immunodeficiency syndromes, acquired':ab,ti OR 'syndrome, acquired immunodeficiency':ab,ti OR 'syndromes, acquired immunodeficiency':ab,ti OR 'aids':ab,ti) AND [2010-2023]/py | HIV/AIDS |
| 2 | ('social networking':ab,ti OR 'networking, social':ab,ti OR 'social networks':ab,ti OR 'network, social':ab,ti OR 'social network':ab,ti OR 'social network analysis':ab,ti OR 'analyses, social network':ab,ti OR 'analysis, social network':ab,ti OR 'network analysis, social':ab,ti OR 'social network analyses':ab,ti OR 'peer':ab,ti OR 'peer influence':ab,ti OR 'peer leader':ab,ti OR 'peer educator':ab,ti OR 'peer mentor':ab,ti OR 'seed':ab,ti OR 'ego':ab,ti OR 'index':ab,ti OR 'index participant':ab,ti OR 'alter':ab,ti OR 'support':ab,ti OR 'partner':ab,ti OR 'Sexual Partners':ab,ti OR 'partner services':ab,ti) AND [2010-2023]/py | Social network |
| 3 | ('hiv self-testing':ab,ti OR 'hiv self-test':ab,ti OR 'hivst':ab,ti OR 'rapid test':ab,ti OR 'home self test':ab,ti OR 'home-based self-test':ab,ti OR 'self-testing':ab,ti OR 'home test':ab,ti OR 'home testing':ab,ti) AND [2010-2023]/py | HIVST |
| 4 | (#1 AND #2 AN D #3) |  |
| **Web of Science (Yield: 623)** | | |
| No. | Terms | Comments |
| 1 | TS=(HIV) OR TS=("Acquired Immunodeficiency Syndrome") OR AB=(HIV OR "human immunodeficiency virus" OR "immunodeficiency virus human" OR "immunodeficiency viruses human" OR "virus human immunodeficiency" OR "viruses human immunodeficiency" OR "human immunodeficiency viruses" OR "human t cell lymphotropic virus type iii" OR "human t cell lymphotropic virus type iii" OR "human t cell leukemia virus type iii" OR "LAV-HTLV-III" OR "lymphadenopathy-associated virus" OR "lymphadenopathy associated virus" OR "Lymphadenopathy-Associated Viruses" OR "virus, lymphadenopathy-associated" OR "viruses, lymphadenopathy-associated" OR "human t lymphotropic virus type iii" OR "aids virus" OR "aids viruses" OR "virus, aids" OR "viruses, aids" OR "acquired immune deficiency syndrome virus" OR "acquired immunodeficiency syndrome virus" OR "HTLV-III" OR "HIV-1" OR "HIV-2" OR "HIV1" OR "HIV2" OR "Acquired Immunodeficiency Syndrome" OR "Immunologic Deficiency Syndrome, Acquired" OR "Acquired Immune Deficiency Syndrome" OR "Acquired Immuno-Deficiency Syndrome" OR "Acquired Immuno Deficiency Syndrome" OR "Acquired Immuno-Deficiency Syndromes" OR "Immuno-Deficiency Syndrome, Acquired" OR "Immuno-Deficiency Syndromes, Acquired" OR "Syndrome, Acquired Immuno-Deficiency" OR "Syndromes, Acquired Immuno-Deficiency" OR "Immunodeficiency Syndrome, Acquired" OR "Acquired Immunodeficiency Syndromes" OR "Immunodeficiency Syndromes, Acquired" OR "Syndrome, Acquired Immunodeficiency" OR "Syndromes, Acquired Immunodeficiency" OR "AIDS") | HIV/AIDS |
| 2 | TS=("Social Networking") OR TS=("Social Network Analysis") OR AB=("Social Networking" OR "Networking, Social" OR "Social networks" OR "network, social" OR "Social network" OR "Social Network Analysis" OR "Analyses, Social Network" OR "Analysis, Social Network" OR "Network Analysis, Social" OR "Social Network Analyses" OR "peer" OR "peer influence" OR "peer leader" OR "peer educator" OR "peer mentor" OR "seed" OR "ego" OR "index" OR "index participant" OR "alter" OR "support" OR "partner" OR "Sexual Partners" OR "partner services") | Social network |
| 3 | AB=("hiv self-testing" OR "hiv self-test" OR "HIVST" OR "home test" OR "rapid test" OR "home self test" OR "home-based self-test" OR "Self-testing" OR "home test" OR "home testing") | HIVST |
| 4 | (#1 AND #2 AND #3) AND (with publication date from Jan 2010 to June 2023) |  |
| **Cochrane Library (Yield: 2424)** | | |
| 1 | HIV or Human Immunodeficiency Virus or Immunodeficiency Virus, Human or Immunodeficiency Viruses, Human or Virus, Human Immunodeficiency or Viruses, Human Immunodeficiency or Human Immunodeficiency Viruses or Human T Cell Lymphotropic Virus Type III or Human T-Cell Lymphotropic Virus Type III or Human T-Cell Leukemia Virus Type III or Human T Cell Leukemia Virus Type III or LAV-HTLV-III or Lymphadenopathy-Associated Virus or Lymphadenopathy Associated Virus or Lymphadenopathy-Associated Viruses or Virus, Lymphadenopathy-Associated or Viruses, Lymphadenopathy-Associated or Human T Lymphotropic Virus Type III or Human T-Lymphotropic Virus Type III or AIDS Virus or AIDS Viruses or Virus, AIDS or Viruses, AIDS or Acquired Immune Deficiency Syndrome Virus or Acquired Immunodeficiency Syndrome Virus or HTLV-III or HIV-1 or HIV-2 or HIV1 or HIV2 or Acquired Immunodeficiency Syndrome or Immunologic Deficiency Syndrome, Acquired or Acquired Immune Deficiency Syndrome or Acquired Immuno-Deficiency Syndrome or Acquired Immuno Deficiency Syndrome or Acquired Immuno-Deficiency Syndromes or Immuno-Deficiency Syndrome, Acquired or Immuno-Deficiency Syndromes, Acquired or Syndrome, Acquired Immuno-Deficiency or Syndromes, Acquired Immuno-Deficiency or Immunodeficiency Syndrome, Acquired or Acquired Immunodeficiency Syndromes or Immunodeficiency Syndromes, Acquired or Syndrome, Acquired Immunodeficiency or Syndromes, Acquired Immunodeficiency or AIDS | HIV/AIDS |
| 2 | Social Networking or Networking, Social or Social Networks or Network, Social or Social Network or Social Network Analysis or Analyses, Social Network or Analysis, Social Network or Network Analysis, Social or Social Network Analyses or peer or peer influence or peer leader or peer educator or peer mentor or seed or ego or index or index participant or alter or support or partner or Sexual Partners or partner services | Social network |
| 3 | hiv self-testing or hiv self-test or HIVST or home test or rapid test or home self test or home-based self-test or self test or Self-testing or home test or home testing | HIVST |
| 4 | (#1 AND #2 AND #3) AND (with publication date from Jan 2010 to June 2023) |  |
| **Wiley (Yield: 51)** | | |
| 1 | HIV OR "human immunodeficiency virus" OR "immunodeficiency virus human" OR "immunodeficiency viruses human" OR "virus human immunodeficiency" OR "viruses human immunodeficiency" OR "human immunodeficiency viruses" OR "human t cell lymphotropic virus type iii" OR "human t cell lymphotropic virus type iii" OR "human t cell leukemia virus type iii" OR "LAV-HTLV-III" OR "lymphadenopathy-associated virus" OR "lymphadenopathy associated virus" OR "Lymphadenopathy-Associated Viruses" OR "virus, lymphadenopathy-associated" OR "viruses, lymphadenopathy-associated" OR "human t lymphotropic virus type iii" OR "aids virus" OR "aids viruses" OR "virus, aids" OR "viruses, aids" OR "acquired immune deficiency syndrome virus" OR "acquired immunodeficiency syndrome virus" OR "HTLV-III" OR "HIV-1" OR "HIV-2" OR "HIV1" OR "HIV2" OR "Acquired Immunodeficiency Syndrome" OR "Immunologic Deficiency Syndrome, Acquired" OR "Acquired Immune Deficiency Syndrome" OR "Acquired Immuno-Deficiency Syndrome" OR "Acquired Immuno Deficiency Syndrome" OR "Acquired Immuno-Deficiency Syndromes" OR "Immuno-Deficiency Syndrome, Acquired" OR "Immuno-Deficiency Syndromes, Acquired" OR "Syndrome, Acquired Immuno-Deficiency" OR "Syndromes, Acquired Immuno-Deficiency" OR "Immunodeficiency Syndrome, Acquired" OR "Acquired Immunodeficiency Syndromes" OR "Immunodeficiency Syndromes, Acquired" OR "Syndrome, Acquired Immunodeficiency" OR "Syndromes, Acquired Immunodeficiency" OR "AIDS" | HIV/AIDS |
| 2 | "Social Networking" OR "Social Network Analysis" OR "Social Networking" OR "Networking, Social" OR "Social networks" OR "network social" OR "Social network" OR "Social Network Analysis" OR "Analyses, Social Network" OR "Analysis, Social Network" OR "Network Analysis, Social" OR "Social Network Analyses" OR "peer" OR "peer influence" OR "peer leader" OR "peer educator" OR "peer mentor" OR "seed" OR "ego" OR "index" OR "index participant" OR "alter" OR "support" OR "partner" OR "Sexual Partners" OR "partner services" | Social network |
| 3 | "hiv self-testing" OR "hiv self-test" OR "HIVST" OR "home test" OR "rapid test" OR "home self test" OR "Self-test" OR "Self-testing" OR "home test" OR "home testing" | HIVST |
| 4 | (#1 AND #2 AN D #3) AND (with publication date from Jan 2010 to June 2023) |  |
