# Supplementary material for: Social network strategies to distribute HIV self‐testing kits: a global systematic review and network meta‐analysis
Source: J Int AIDS Soc. 2024 Jul 24;27(7):e26342. doi: 10.1002/jia2.26342 (PMC11269052; doi:10.1002/jia2.26342)
Supplement: Supplementary file 2 — Table S1(a): HIVST distribution strategy details – No comparison group Table S1(b): HIVST distribution strategy details – Comparison group Table S2: Outcomes of the included studies Table S3(a): Network meta‐analysis relative effects (league) table of HIVST distribution strategies – HIV testing uptake Table S3(b): Network meta‐analysis ranking probabilities of HIVST distribution strategies – HIV testing uptake Table S4: Linkage to ART or Any Care Among people living with HIV by Distribution Strategy, Study Design and Population Subgroup Table S5: Risk of bias for included studies – Observational studies Table S6: Risk of bias for included studies–RCTs and a quasi‐experimental study [file JIA2-27-e26342-s002.docx]

Table of Contents

[S1(a) Table: HIVST distribution strategy details - No comparison group 2](#_Toc148025724)

[S1(b) Table: HIVST distribution strategy details - Comparison group 6](#_Toc148025725)

[S2 Table: Outcomes of the included studies 19](#_Toc148025726)

[S3(a)Table: Network meta-analysis relative effects (league) table of HIVST distribution strategies - HIV testing uptake 23](#_Toc148025727)

[S3(b)Table: Network meta-analysis ranking probabilities of HIVST distribution strategies - HIV testing uptake 24](#_Toc148025728)

[S4 Table: Linkage to ART or Any Care Among people living with HIV by Distribution Strategy, Study Design and Population Subgroup 25](#_Toc148025729)

[S5 Table: Risk of bias for included studies - Observational studies 26](#_Toc148025730)

[S6 Table: Risk of bias for included studies – RCTs and a quasi-experimental study 27](#_Toc148025731)

[S1 Figure: Risk of bias for included studies – RCTs and a quasi-experimental study 28](#_Toc148025732)

## S1(a) Table: HIVST distribution strategy details - No comparison group

| **Study** | **Seed size** | **Social network**  **member size** | **Method of identifying seeds** | **Method of identifying network members** | **Role of social network** | **HIVST Delivery Method** | **Who distributed HIVST** | **Where HIVST delivered** | **How HIVST delivered** | **Testing choice offered** | **Testing support** | **Additional intervention components** |
| --- | --- | --- | --- | --- | --- | --- | --- | --- | --- | --- | --- | --- |
| Kitenge 2022 | 640 | 449 | At PHCs, lay counsellors invited all users to participate. At community-based testing sites, CHWs introduced the study to all individuals visiting these sites. | PRs distributed HIVST kits to their sexual partner, family members or anyone in their social network who was above 18 years old. | Accessing the hard to-reach & Diffusion | Secondary | Lay counsellors or community health workers to peer to social network | Social network | In person | No | Instruction on use | No |
| Kwan 2023 | 150 | 313 | Seed participants were recruited from various internet platforms, including social media platforms, a web-based forum, and location-based networking apps used by MSM. | Seed MSM participants invited their peers. | Accessing the hard to-reach & Diffusion | Secondary | Web-based platform to participants to social network | Online | Mail | Yes (finger-prick or oral fluid test) | Real-time support, including in-person, video call, and instant messaging support, at kit request | Monetary incentive (To encourage return, an incentive of HK $20 (US $2.56) was given after manual verification of the result. ) |
| Lippman 2018 | 127 | 728 | Participants were recruited from all HIV-negative MpMS. | Participants shared the kits with sexual partners and others with whom they felt safe distributing kits. | Accessing the hard to-reach & Diffusion | Secondary | HCW to participants to social network | social network | In person | HIVST (oral fluid or blood) | All participants were shown a demonstration on how to use both the oral fluid and fingerstick HIV self-tests. | No |
| Li S 2021 | 1422 | 394 | 6 key opinion leaders of MSM disseminated an HIVST recruitment advertisement to invite MSMs. | IPs shared the HIVST service links on social media with their sexual partners and friends. | Accessing the hard to-reach & Diffusion | Online & mail | HIVST application link for phone app to index to alter | Online | Mail | No | HIV posttest consultation | Monetary incentive (US $2 was paid to participants who uploaded their test results) |
| Matovu 2020 | 34 | 298 | Community residents selected one peer-leader per social network grouping. | Peer-leaders distributed kits to eligible social network members. | Promote HIVST and increase linkage to HIV care among newly diagnosed HIV-positive individuals | Community-based | HCW to Peer-educators to social network | In community | In person | No | Peer-leaders received a training on oral HIV self-testing processes; basic counselling, communication and referral skills; and how to approach social network members at the time of distributing the kits;a practical demonstration of how the HIV self-testing exercise is conducted | No |
| Nasuuna 2022 | 9378 | 9378 | Recruitment of KP peers and pregnant and lactating mothers. | HIVST was implemented using peer-to-peer model for KPs and secondary distribution for partners of consenting pregnant and lactating mothers. | Accessing the hard to-reach & Diffusion | Secondary | peer-to-peer/HCW to participants to partner | In community | In person | No | 3-day trainings prior to distribution of HIVST kits. Education on how to use HIVST kits was provided in the local language | Phone call (Mothers and peers were asked to confirm delivery of the kits to recipients through a phone call.) |
| Nguyen 2019 | - | - | KPs and their partners were offered HIVST by peer-educators. | Community-led outreach and social networks were used to promote HIVST. | Promote HIVST and follow-up with self-testers | Community-based | HCW to Peer-educators to KP and their partners | In community | In person | Self-testers were given the choice to test with or without assistance. | Assisted HIVST | No |
| Nguyen 2019 | - | - | KPs and their partners were offered HIVST by peer-educators. | Both in‐person and social network methods were used to mobilize key populations to test for HIV and offer HTS to partners of people living with HIV. | mobilize key populations to test for HIV and offer HTS to partners of people living with HIV | Community-based | HCW to Peer-educators to KP and their partners | In community | In person | Yes: lay provider‐delivered rapid testing / HIVST | Peer educators were trained to conduct rapid testing, perform and demonstrate self‐testing, provide pre‐test information and post‐test counselling | No |
| Pintye 2019 | 362 | 343 | 8 purposively selected PrIYA sites where MCH and FP clients seeking routine services are offered multiple HIVSTs for at-home male partner and couples HIV testing. | Participants were encouraged to offer an HIVST to their male partner and to use self-tests together as a couple if they felt comfortable doing so. | Increased coverage of HIV testing services for partners and the odds of partners being linked to care or prevention | Secondary | HCW to participants to partner | Partners among HIV-uninfected women | In person | No | Instructions and demonstration of how to use and telephone support | No |
| Thirumurthy 2016 | 280 | 445 | HIV-uninfected women aged 18–39 years were recruited at a health facility with antenatal (ANC) and postpartum (PPC) clinics, and a drop-in center for female sex workers (FSW). | Male partners of HIV-uninfected women. | Increased coverage of HIV testing services for partners and the odds of partners being linked to care or prevention | Secondary | HCW to women with high HIV incidence to their sexual partners | Partners among HIV-uninfected women | In person | No | Instructions on using the OraQuick Rapid HIV 1/2 Test and telephone support | No |
| Wu D 2021 | 371 | 281 | MSMs were recruited from a social-media based online system. | Index MSMs distributed the kits to other social contacts, including partners or friends. | Accessing the hard to-reach & Diffusion | Secondary | HIVST application link for phone app to index to alter | Social network | Mail | No | Instruction on use and HIV post-test consultation | Monetary incentive (US$3 was provided to all participants who completed a questionnaire.) |
| Zhang J 2021 | 471 | 180 | Participants were recruited from MSM in the ongoing PrEP pragmatic trial. | Participants shared HIVST with their male sexual partners. | Increased coverage of HIV testing services for partners and the odds of partners being linked to care or prevention | Secondary | HCW to participants to partner | partners among MSM | In person | No | A service account on WeChat provided web-based services on the application of extra testing kits, instructions on self-testing, real-time consultation with the staff, uploading of test outcomes, and follow-up questionnaires | Daily or on-demand PrEP |
| Zishiri 2022 | ANC:4235  index:1649 | ANC:3475  index:1312 | ANC attendees and people or those newly diagnosed with HIV clients were given HIVST kits. | Clinic attendees distributed HIVST kits to their partners. | Increased coverage of HIV testing services for partners and the odds of partners being linked to care or prevention | Secondary | HCW to participants to partner | In community | In person | No | Instructions and demonstration of how to use | No |

Abbreviations: ANC, antenatal care clinic; FSW, female sex workers; HCW, healthcare worker; HIV, human immunodeficiency virus; HIVST, HIV self-testing; HTS, HIV testing services; MSM, men who have sex with men; PrEP, pre-exposure prophylaxis.

## S1(b) Table: HIVST distribution strategy details - Comparison group

| **Study** | **Seed size** | **Social network**  **member size** | **Method of identifying seeds** | **Method of identifying network members** | **Randomization method** | **Role of social network** | **HIVST Delivery Method** | **Who distributed HIVST** | **How HIVST delivered** | **Testing choice offered** | **Testing support** | **Additional intervention components** | **Comparison** |
| --- | --- | --- | --- | --- | --- | --- | --- | --- | --- | --- | --- | --- | --- |
| Chanda 2017 | 160  (Standard:53, Delivery: 53, Coupon: 54) | 965  (Standard:320, Delivery: 316, Coupon: 329) | Recruitment of current or former FSWs as peer educators. | Participants were recruited by peer educators. | Peer educator–participant groups were randomized as clusters in a 1:1:1 fashion to 1 of the 3 study arms. | Diffusion & Impact on risk and health behaviors | Facility & secondary by peer | HCW to Peer-educators to participants | In person | No | Instructions for use and telephone support | NA | Routine facility-based HIV testing |
| Choko 2019 | 2349  (I:1941, C:408) | 2349  (I:1941, C:408) | Women aged 18 years and older attending an ANC at primary care clinics, whose primary male partner was not known to be on ART were recruited. | Women were given an invitation letter addressed to their male partner informing them of the importance of having an HIV test. | We block randomized ANC days (representing clusters of women) to 1 of 6 trial arms (standard of care [SOC] and 5 intervention arms; ratio 1:1:1:1:1:1). | Increased coverage of HIV testing services for men and the odds of men being linked to care or prevention | Secondary | HCW to participants to partners | In person | No | Instructions for use | Monetary incentive ($3, $10, and a phone reminder) | Routine facility-based HIV testing |
| Choko 2021 | ANC:4544  (SOC:1447, HIVST:1465, HIVST plus:1632)  index:708  (SOC:234, HIVST:169, HIVST plus:305) | ANC:2604 (SOC:498, HIVST: 1106, HIVST plus:1000)  index:649 (SOC:209, HIVST:155, HIVST plus:285) | Eligible women attending ANC were enrolled into the ANC clinic cohort and people newly diagnosed with HIV during routine clinic HIV testing were enrolled into the index cohort. | Eligible women provided with materials and brief training for their male partner. Index patients delivered materials to all sexual contacts over the past 12 months. | Restricted randomization (blocking) was used to randomize the 27 clusters to three arms in a ratio of 1:1:1, with district and HIV prevalence in ANC as the variables for restriction. | Accessing the hard to-reach & Diffusion | Secondary | HCW to participants to partner | In person | No | Instructions and demonstration of how to use | Monetary incentive | Routine facility-based HIV testing |
| Dovel 2019 | 484  (I:349, C:135) | 365  ((I:258, C:107) | Individuals living with HIV and on ART were recruited during routine ART clinic visits. | Partners of ART clients. | Computer-generated randomization was used to assign clients to either the PRS or HIVST arms in a ratio of 1:2·5, respectively. | Increased coverage of HIV testing services for partners and the odds of partners being linked to care or prevention | Secondary | HCW to participants to partner | In person | No | Instructions and demonstration of how to use | NA | Routine facility-based HIV testing |
| Frye 2021 | 188  (I:89, C:99) | 188  (I:90, C:98) | Recruitment was conducted via online advertising, face-to face outreach and referrals by study participants. | Participants completed an online contact card and sent a link to a friend. | Participants were randomized as friend pairs in a 1:1 ratio into either the TRUST intervention or the time- and attention-matched control intervention arm using assignments generated by the study data analyst using Sealed Envelope Ltd. | Impact on risk and health behaviors. TRUST was designed to increase uptake of consistent (every three months) HST among YBMSM/TW in New York City. | Direct | HCW to participants | In person or Mail-in | No | Instructions for use and peer-based behavioral intervention | Monetary incentive | Time and attention control arm |
| Gichangi 2018 | 1410  (I:472, C:938) | 1133  (I:396, C:737) | The health facility nurse identified women attending first ANC and referred them to the trained study nurse. | Partner distribution in community. | Participants were individually randomized into 1 of the 3 study arms. | Increased coverage of HIV testing services for men and the odds of men being linked to care or prevention | Secondary | HCW to participants to partners | In person | No | Instructions for use | Advise on partner negotiation and communication | Standard information card to invite male partners to the clinic for routine or HIV care |
| Joseph 2022 | 176  (I:85, C:91) | 176  (I:85, C:91) | Recruitment of WLHIV who were attending HTS, HIV care/treatment services or antenatal care services by the research team and clinic staff. | Male partners of women living with HIV. | Participants were randomized 1:1 using a random number table by participant identifier (ID). | Increased coverage of HIV testing services for men and the odds of men being linked to care or prevention | Secondary | HCW to participants to partner | In person | No | Instructions for use and telephone support | NA | Routine facility-based HIV testing |
| Korte 2020 | 1514  (I:777, C:737) | 1434 (I:735,  C: 699) | Pregnant women aged 14 years or older and either HIV-positive or HIV-negative who were attending ANC at any time during their pregnancy were recruited. | Women to encourage their partners to test. | We used simple randomization to assign each study day to intervention or control, and this random assignment was the same each day for all 3 study sites. Across all study sites, to avoid contamination between arms, study days were randomized to arm 1 or 2. | Increased coverage of HIV testing services for men and the odds of men being linked to care or prevention | Secondary | HCW to participants to partner | In person | No | Instruction enhancement, video or study hotline | NA | Routine facility-based HIV testing |
| Lightfoot 2018 | 30 | 165 | The peer recruiters were identified from HIV-related support groups, local gay bars, online social networking and dating apps, community-based organizations, and word of mouth. | The peer recruiters distributed OraQuick oral fluid HIV test kits to friends who they believed were AAMSM or LMSM, age 18–45, and had not tested in the last year. | N/A | Peers, who were most effective at reaching infrequent, and non-testers. | Secondary | HCW to Peers to social network | In person | No | Instructions for use | Monetary incentive(Each peer received $150 after distribution of all 5 HIVST tests. Testers had the alternative option to provide contact information for receipt of cash or another gift card for $25. | Testers in County Testing Program |
| MacGowan 2020 | 667 | 2301 | Persons who clicked the ads on social network, music, and dating websites were directed to research sites to complete eligibility screening. | Distribution HIV self-tests to social network members. | Participants were randomly assigned to the self-testing (ST) arm or control arm using a computer-generated stochastic random 1:1 allocation. | Accessing the hard to-reach & Diffusion | Secondary | Website online to index to alter | mail | No | Instruction enhancement, video or study hotline | NA | Website with routine HIV testing information |
| Marwa 2019 | 809  (C:387, I:422) | 758  (C:362, I:396) | Pregnant women attending antenatal clinics were recruited in 14 clinics in the eastern and central regions of Kenya. | Male partners of ANC client were invited. | Randomization of participants to the study arms was at a 1:1:1 ratio, using a computer-generated randomization list. | Increased coverage of HIV testing services for men and the odds of men being linked to care or prevention | Secondary | HCW to participants to partners | In person | No | Instruction and consultation on partner testing in general | NA | Invitation letter for clinic-based HIV testing |
| Masters 2016 | 570  (I:284, C:286) | 570  (I:284, C:286) | Trained research assistants screened and enrolled women seeking ANC or PPC at the three facilities, in a private location away from regular clinic activities. | Partner distribution in community. | Participants were randomized in a 1:1 ratio using balanced block randomization (block size 20) to an HIVST group or a comparison group. | Increased coverage of HIV testing services for men and the odds of men being linked to care or prevention | Secondary | HCW to participants to partners | In person | No | Instructions and demonstration of how to use | Advise on partner negotiation and communication | Invitation card for clinic-based HIV testing |
| Merchant 2018 | 88  (I:51, C:37) | 88  (I:51, C:37) | Via multiple social media platforms. | YMSMs recommended three other 18–24-year-old black, Hispanic, or white YMSM. | Within each racial/ethnic group, we randomly assigned participants into one of three study arms (1:1:1 randomization) using block sizes of six. | Accessing the hard to-reach & Diffusion | Online & mail | Website/ Study app to participants to social network | Mail | No | No | Monetary incentive (Participants received a $10 internet-based gift card for completing the follow-up questionnaire, and up to three $5 gift cards for providing email addresses of other YMSM to be contacted about the study. ) | Routine facility-based HIV testing |
| Mujugira 2022 | 489  (I:328, C:161) | 234  (I:159, C:75) | PWLHIV attending antenatal care who reported that their partner's HIV status was unknown. | Secondary distribution of HIV self-test kits (HIVST) from HIV-negative pregnant women to their partners. | Eligible participants were randomized 2:1 to HIVST secondary distribution or an invitation for fast-track HIV testing for their partner. | Increased coverage of HIV testing services for men and the odds of men being linked to care or prevention | Secondary | HCW to participants to partner | In person | No | trained in the use and interpretation of HIVST | NA | Routine facility-based HIV testing |
| Okoboi 2020 | 15 | 150 | Fifteen MSM peers who trained in HIVST testing procedures and results interpretation. | Peers distributed HIVST kits to MSM in their social and sexual networks. | N/A | Increasing testing coverage among non-testers. | Secondary | HCW to Peers to social network | In person | No | Instructions and demonstration of how to use and telephone support | Transport reimbursement and T-shirts, backpacks and umbrellas as tokens of appreciation | Routine facility-based HIV testing |
| Ortblad 2017 | 120  (Direct: 37, Facility: 42, Standard: 41) | 840  (Direct: 259, Facility: 294, Standard: 287) | Recruitment of FSW peer educators. | Peer-educators recruitment, research assistants telephone screening, eligibility assessment, and enrollment. | We randomized FSW peer educator groups 1:1:1 to 1 of 3 study arms. | Have good access to other FSWs and are able to engage with FSWs who do not normally utilize the health system & Diffusion & Impact on risk and health behaviors | Facility & secondary by peer | HCW to Peer-educators to participants | In person | No | Instructions for use and telephone support | NA | Routine facility-based HIV testing |
| Pettifor 2020 | 287  (I:141, C:146) | 1107  (I:701, C:406) | Recruitment of young women from Agincourt Health and Social Demographic Surveillance System (AHDSS). | Young women offered test kits/invitations to peers and sexual partners. | Young women ages 18-26 were randomized using block randomization with a 1:1 allocation to either. | Secondary distribution of HIV self-test kits is one way to reach populations who test less frequently and hard-to-reach groups. | Secondary | HCW to index to alter | In person | Yes (choice of either a clinic-based HCT invitation or oral HIV Self-Testing (HIVST) kits.) | Instructions for use, include pre- and post-test counseling information and a “frequently asked questions” document on HIV self-testing | NA | Routine facility-based HIV testing |
| Sha 2022 | 154  (I:92, C:62) | 205  (I:179, C:26) | Community volunteers or public health workers invited men who visited the three clinics for HIV testing. | Indexes distributed HIV/syphilis dual self-testing kits to people in their social networks. | The two arms were implemented one at a time to compare the intended outcomes in similar catchment areas without having people choose between the two. | Accessing the hard to-reach & Diffusion | Secondary | HCW to index to alter | In person | No | Instructions for use | Monetary incentive (Indexes received $3 after completing the baseline survey and $5 for a follow-up survey. Alters and corresponding indexes both received an additional $3 when alters uploaded their test results.) | Testing card referral (facility-based test) |
| Shahmanesh 2021 | 57  (I: 38, C: 19) | 4163  (I: 3065, C: 1098) | Recruitment of 24 pairs of peer navigators through local municipal and traditional leaders. | Peer navigators recruited young people in community settings near schools and homes. | The final groupings of peer navigators into three arms were completed using statistical software, into three groups of 8 pairs and three floating peer navigators (A, B and C). | Diffusion & Impact on risk and health behaviors | Facility & secondary by peer | HCW to Peer-educators to participants | In person | No | In all three arms, peer navigators promoted sexual health and the benefits of HIV testing PrEP and ART. In both intervention arms, they also demonstrated how to use the HIVST kit. | Monetary incentive | Routine facility-based HIV testing |
| Thirumurthy 2021 | 2090  (I:1057, C:1033) | 2090  (I:1057, C:1033) | We prepared a list of women aged ≥18 years who were potentially eligible within each cluster. | We encouraged participants to use the tests themselves and to offer tests to their primary sexual partner and to any other sexual partners with whom unprotected sex was likely. | Randomization with a computer-generated algorithm. | Increased coverage of HIV testing services for men and the odds of men being linked to care or prevention | Secondary | HCW to participants to partners | In person | No | Instructions for use, include information on interpretation of results and a list of nearby clinics where users could confirm their test result and seek post-test services. | Participants were asked to contact study staff by phone to request additional self-tests during interim periods. | Invitation card for clinic-based HIV testing |
| Wango 2023 | 324  (I:161, C:163) | 324  (I:161, C:163) | Through multiple approaches: the Determined, Resilient, Empowered, AIDS-Free, Mentored, Safe program; community health volunteer outreach; recruitment posters; village elders who identified households with AGs; and government youth employment programs. | Participants were given three months to offer the self-test/referral coupon to their partner. | Participants were randomly assigned to the self-testing (ST) arm or control arm using a computer-generated stochastic random 1:1 allocation. | Increased coverage of HIV testing services for men and the odds of men being linked to care or prevention | Secondary | HCW to participants to partners | In person | No | Trained on how to perform the self-test and a list of health facilities where partners could access confirmatory testing and HIV care services | NA | Referral coupon for facility-based testing |
| Young 2013 | 16  (I:8, C:8) | 112  (I:55, C:57) | 18 peer leaders were recruited from community organizations serving African American and Latino MSM. | Participants were recruited from ads on Internet and social networking sites. | Participants were randomly and blindly assigned to 1 of 2 intervention or control groups and then randomly assigned to 2 peer leaders within that group. | Diffusion & Impact on risk and health behaviors, to increase HIV prevention and testing behaviors by changing social norms. | Direct | HCW to Peer-educators to participants | Mail-in | No | Instructions for use | NA | General health information |
| Young 2022 | 79  (I:79, C:0) | 900  ((I:450, C:450) | 79 peer leaders were recruited with help from community organizations serving Latinx and African American MSM. | From online advertisements on Facebook, Craigslist, and other websites/applications; community physical venues frequented by Latinx and African American MSM; and from direct referrals from study participants. | Using a random number generator with participants blinded to assignment and unable to request group or condition assignment. | Diffusion & Impact on risk and health behaviors, to increase HIV prevention and testing behaviors by changing social norms. | Direct | HCW to Peer-educators to participants | Mail-in | No | Instructions for use and communicate in the online community, by sending messages, chats, and wall posts | NA | Non-peer-led HIVST |
| Van Der Elst 2017 | 11  (I:6, C:5) | 1027  (I:337, C:690) | Mobilizations were done through GBMSM peers from a local GBMSM-led community group. | Gay, bisexual, and other MSM  peers mobilized in community. | NA | Accessing the hard to-reach & Diffusion | Secondary | HCW to Peers to social network | In person | No | Close supervision and daily feedback | Participants asked to report for confirmatory HTC at the clinic | Routine facility-based HIV testing |
| Zhang 2020 | 207  (I:105, C:102) | 429  (I:291, C:138) | Through collaborating with Zuo An Cai Hong, a gay-friendly CBO located in Changsha with satellite offices in the other 3 study cities, we recruited MSM participants via community outreach, social media, and hotlines. | Partner distribution in community. | Two separate randomized number tables for recent testers and non-recent testers were generated with SPSS V.18.0 and used to divide participants randomly (at 1:1 allocation ratio) into the intervention and control arms. | Increased coverage of HIV testing services for sexual partners and the odds of sexual partners being linked to care or prevention | Secondary | HCW to participants to partners | In person | No | Detailed electronic users’ instructions and counseling information, including 24/7 hotlines and an official WeChat study account to obtain consultation | Participants sent an electronic photocopy of their test result via secured individual WeChat contact after they used each HIVST kit, and then they were replenished with new free kits | Routine facility-based HIV testing |
| Zhou 2022 | 309  (C:102, SD-M:103, SD-M-PR:104) | 344  (C:58, SD-M:101, SD-M-R:185) | Volunteers of Xutong enrolled participants by advertising the recruitment and trial introduction in the WeChat public platform. | Index participants distributed HIVST kits to members within their social networks. | Eligible participants were randomly assigned to one of the 3 arms individually and independently by a computer-generated program electronically. | Accessing the hard to-reach & Diffusion | Secondary | HCW to index to alter | Mail-in | No | Instructions for use | Monetary incentive (Index participants in the 2 intervention groups could receive a fixed incentive ($3 USD) online for the verified test result uploaded to the digital platform by each unique alter.) | Standard secondary distribution |

Abbreviations: ANC, antenatal care clinic; ART, antiretroviral therapy; FSW, female sex workers; HCW, healthcare worker; HIV, human immunodeficiency virus; HIVST, HIV self-testing; HTS, HIV testing services; MSM, men who have sex with men; PrEP, pre-exposure prophylaxis; SOC, standard of care.

## S2 Table: Outcomes of the included studies

| **Study** | **Study arm** | **HIV testing uptake** | | | **HIV prevalence** | | | **Linkage to ART or HIV care among HIV positive** | | | |
| --- | --- | --- | --- | --- | --- | --- | --- | --- | --- | --- | --- |
|  |  | **Time point** | **Offered HIVST** | **Tested for HIVST** | **Time point** | **All positive** | **Tested for HIVST** | **Time point** | **ART initiation** | **Linked to HIV care** | **HIV positive** |
| Chanda 2017 | Standard of care | 2016.9-2017.2 | 1month: 296, 4months: 301 | 1month: 262, 4months: 226 | 2016.9-2017.2 | 1month: 59, 4months: 84 | 1month: 288, 4months: 298 | 2016.9-2017.2 | 1month: 27, 4months: 54 | 1month: 44, 4months: 72 | 1month: 59, 4months: 84 |
| Chanda 2017 | Delivery | 2016.9-2017.2 | 1month: 296, 4months: 295 | 1month: 280, 4months: 248 | 2016.9-2017.2 | 1month: 49, 4months: 74 | 1month: 294, 4months: 292 | 2016.9-2017.2 | 1month: 11, 4months: 35 | 1month: 25, 4months: 53 | 1month: 49, 4months: 74 |
| Chanda 2017 | Coupon | 2016.9-2017.2 | 1month: 294, 4months: 302 | 1month: 248, 4months: 241 | 2016.9-2017.2 | 1month: 36, 4months: 77 | 1month: 291, 4months: 300 | 2016.9-2017.2 | 1month: 9, 4months: 44 | 1month: 19, 4months: 59 | 1month: 36, 4months: 77 |
| Choko 2019 | Standard of care | 28 days | 408 | 71 | 28 days | 3 | 71 | 28 days | 3 | - | 3 |
| Choko 2019 | Partner distribution in community | 28 days | 1941 | 1801 | 28 days | 43 | 1801 | 28 days | 39 | - | 43 |
| Choko 2021 | Standard of care | 28 days | ANC:1447 index:234 | ANC:498 index:209 | 28 days | index:9 ANC:2 | index:209 | - | ANC:2 | - | index:9 ANC:2 |
| Choko 2021 | HIVST | 28 days | ANC:1465 index:169 | ANC:1106 index:155 | 28 days | index:13 ANC:0 | index:155 | - | ANC:0 | - | index:13 ANC:0 |
| Choko 2021 | HIVST plus | 28 days | ANC:1632 index:305 | ANC:1000 index:285 | 28 days | index:32 ANC:22 | index:285 | - | ANC:22 | - | index:32 ANC:22 |
| Dovel 2019 | Standard of care | 2018.3-2018.6 | 107 | 27 | - | 4 | 27 | 12 months | 3 | - | 4 |
| Dovel 2019 | Partner distribution in community | 2018.3-2018.6 | 258 | 183 | - | 30 | 183 | 12 months | 14 | - | 30 |
| Frye 2021 | Standard of care | 2016.7-2019.1 | 197 | 3 months: 83  6 months: 83 | - | - | - | - | - | - | - |
| Frye 2021 | Peer-based behavioral intervention | 2016.7-2019.1 | 179 | 3 months: 102 6 months: 97 | - | - | - | - | - | - | - |
| Gichangi 2018 | Standard of care | 3 months | 938 | 239 | - | - | - | - | - | - | - |
| Gichangi 2018 | Partner distribution in community | 3 months | 472 | 322 | - | - | - | - | - | - | - |
| Joseph 2022 | Standard of care | 3 months | 85 | 44 | 3 months | 6 | 44 | 3 months | 6 | - | 6 |
| Joseph 2022 | Partner distribution in community | 3 months | 91 | 66 | 3 months | 9 | 66 | 3 months | 6 | - | 9 |
| Korte 2020 | Standard of care | 3 months | 699 | 187 | 3 months | 10 | 187 | 3 months | - | 4 | 6 |
| Korte 2020 | Partner distribution in community | 3 months | 735 | 519 | 3 months | 34 | 519 | 3 months | - | 6 | 26 |
| Lightfoot 2018 | Self-Testers | 2016.1-2017.3 | 165 | 114 | 2016.1-2017.3 | 7 | 114 | - | - | - | 7 |
| Lightfoot 2018 | Testers in County Testing Program | 2016.1-2017.3 | 3483 | 1205 | 2016.1-2017.3 | 18 | 1205 | - | - | - | 18 |
| MacGowan 2020 | Standard of care | 2015.3-2016.11 | - | - | 2015.3-2016.11 | - | - | - | - | - | - |
| MacGowan 2020 | Peer distribution in community | 2015.3-2016.11 | 2864 | 2301 | 2015.3-2016.11 | 34 | 2301 | - | - | 26 | 36 |
| Marwa 2019 | Standard of care | 3 months | 362 | 133 | - | - | - | - | - | - | - |
| Marwa 2019 | Partner distribution in community | 3 months | 396 | 327 | - | - | - | - | - | - | - |
| Masters 2016 | Standard of care | 3 months | 286 | 148 | 3 months | 4 | 148 | 3 months | - | 3 | 4 |
| Masters 2016 | Partner distribution in community | 3 months | 284 | 258 | 3 months | 8 | 258 | 3 months | - | 2 | 8 |
| Merchant 2018 | Standard of care | 3 months | 71 | 10 | - | - | - | - | - | - | - |
| Merchant 2018 | mail-testing | 3 months | 57 | 14 | - | - | - | - | - | - | - |
| Merchant 2018 | Online & mail distribution | 3 months | 82 | 17 | - | - | - | - | - | - | - |
| Mujugira 2022 | Standard of care | 12 months | 161 | 75 | 12 months | 13 | 75 | 12 months | 10 | - | 13 |
| Mujugira 2022 | Partner distribution in community | 12 months | 328 | 159 | 12 months | 36 | 159 | 12 months | 25 | - | 36 |
| Okoboi 2020 | Standard of care | 2018.6-2018.8 | - | 147 | 2018.1-2018.3 | 4 | 147 | - | - | - | 4 |
| Okoboi 2020 | Peer community distribution | 2018.6-2018.8 | 150 | 143 | 2018.6-2018.8 | 8 | 143 | - | 8 | 8 | 8 |
| Ortblad 2017 | Peer educator community distribution HIVST | 2016.10-2017.3 | 1month: 289, 4months: 262 | 1month: 275, 4months: 261 | 2016.10-2017.3 | 1month: 39, 4months: 44 | 1month: 287, 4months: 260 | 2016.10-2017.3 | 1month: 13, 4months: 19 | 1month: 17, 4months: 27 | 1month: 39, 4months: 44 |
| Ortblad 2017 | HCW at health facility | 2016.10-2017.3 | 1month: 321, 4months: 297 | 1month: 258, 4months: 288 | 2016.10-2017.3 | 1month: 54, 4months: 80 | 1month: 312, 4months: 289 | 2016.10-2017.3 | 1month: 10, 4months: 27 | 1month: 13, 4months: 37 | 1month: 54, 4months: 80 |
| Ortblad 2017 | Standard-of-care | 2016.10-2017.3 | 1month: 316, 4months: 302 | 1month: 226, 4months: 263 | 2016.10-2017.3 | 1month: 39, 4months: 53 | 1month: 301, 4months: 294 | 2016.10-2017.3 | 1month: 13, 4months: 24 | 1month: 25, 4months: 37 | 1month: 39, 4months: 53 |
| Pettifor 2020 | Peer community distribution | 3 months | 373 | 119 | 3 months | 4 | 119 | - | - | - | 4 |
| Pettifor 2020 | Standard of care | 9 months | 406 | 114 | 9 months | 8 | 114 | - | - | - | 8 |
| Pettifor 2020 | Peer community distribution | 9 months | 701 | 393 | 9 months | 14 | 393 | - | - | - | 14 |
| Sha 2022 | Secondary distribution | 2019.5-2020.1 | 179 | 139 | 2019.5-2020.1 | 8 | 139 | - | - | - | 8 |
| Sha 2022 | Testing card referral | 2019.10-2020.1 | 26 | 1 | 2019.10-2020.1 | 0 | 1 | - | - | - | 0 |
| Shahmanesh 2021 | Standard of care | 2019.3-2019.9 | 1098 | - | - | - | - | - | - | 111 | - |
| Shahmanesh 2021 | Direct HIVST distribution | 2019.3-2019.9 | 1480 | - | - | - | - | - | - | 111 | - |
| Shahmanesh 2021 | Peer community distribution | 2019.3-2019.9 | 1585 | - | - | - | - | - | - | 50 | - |
| Thirumurthy 2021 | Standard of care | 2017.6-2020.3 | 6months: 915, 24months:1033 | 6months: 422, 24months:985 | 2017.6-2020.3 | 132 | 985 | - | - | - | - |
| Thirumurthy 2021 | Partner distribution in community | 2017.6-2020.3 | 6months: 921, 24months:1057 | 6months: 806, 24months:1017 | 2017.6-2020.3 | 261 | 1017 | - | - | - | - |
| Wango 2023 | Standard of care | 2021.4-2021.7 | 161 | 119 | 2021.4-2021.7 | 0 | 119 | 2021.4-2021.7 | - | 0 | 0 |
| Wango 2023 | Partner distribution in community | 2021.4-2021.7 | 163 | 153 | 2021.4-2021.7 | 2 | 153 | 2021.4-2021.7 | - | 1 | 2 |
| Young 2013 | Standard of care | 2011.3-2011.6 | 11 | 2 | - | - | - | - | - | - | - |
| Young 2013 | Peer-leaders distribution in community | 2011.3-2011.6 | 25 | 9 | - | - | - | - | - | - | - |
| Young 2022 | Standard of care | 2017.2-2021.1 | 450 | 102 | - | - | - | - | - | - | - |
| Young 2022 | Peer-leaders distribution in community | 2017.2-2021.1 | 450 | 130 | - | - | - | - | - | - | - |
| Van Der Elst 2017 | Standard of care | - | - | - | 2015.7-2015.12 | 24 | 690 | 1 Day(s) | 20 | - | 24 |
| Van Der Elst 2017 | Peer community distribution HIVST | - | - | - | 2016.3-2016.6 | 29 | 337 | 14 Day(s) | 24 | - | 29 |
| Zhang 2020 | Standard of care | 2018.4-2018.6 | 251 | 138 | 2018.4-2018.6 | 0 | 138 | 2018.4-2018.6 | - | - | - |
| Zhang 2020 | Partner distribution in community | 2018.4-2018.6 | 368 | 291 | 2018.4-2018.6 | 8 | 291 | 2018.4-2018.6 | - | 8 | 8 |
| Zhou 2022 | Control | 2019.10-2020.12 | 65 | 58 | - | 6 | 58 | - | - | - | 6 |
| Zhou 2022 | SD-M | 2019.10-2020.12 | 107 | 101 | - | 4 | 101 | - | - | - | 4 |
| Zhou 2022 | SD-M-PR | 2019.10-2020.12 | 187 | 185 | - | 5 | 185 | - | - | - | 5 |

HIV testing uptake among all randomized or enrolled; HIV positivity among HIV tested; Linkage to ART or HIV care among HIV positive.

Abbreviations: ANC, antenatal care clinic; ART, antiretroviral therapy; HCW, healthcare worker; HIV, human immunodeficiency virus; HIVST, HIV self-testing.

## S3(a)Table: Network meta-analysis relative effects (league) table of HIVST distribution strategies - HIV testing uptake

|  | **Facility-based testing** | **Peer distribution** | **Partner distribution** | **Peer educator distribution** |
| --- | --- | --- | --- | --- |
| **Facility-based testing** | **Facility-based testing** | 2.58 (1.53, 4.60) | 1.91 (1.46, 2.51) | 1.22 (0.75, 1.98) |
| **Peer distribution** | 0.39 (0.22, 0.66) | **Peer distribution** | 0.74 (0.39, 1.33) | 0.47 (0.22, 0.96) |
| **Partner distribution** | 0.52 (0.40, 0.68) | 1.35 (0.75, 2.57) | **Partner distribution** | 0.64 (0.37, 1.11) |
| **Peer educator distribution** | 0.82 (0.51, 1.33) | 2.12 (1.05, 4.56) | 1.57 (0.90, 2.73) | **Peer educator distribution** |

## S3(b)Table: Network meta-analysis ranking probabilities of HIVST distribution strategies - HIV testing uptake

| **Testing and distribution strategy** | **Probability of ranking 1** | **Probability of ranking 2** | **Probability of ranking 3** | **Probability of ranking 4** |
| --- | --- | --- | --- | --- |
| **Facility-based testing** | 0.00 | 0.00 | 0.20 | 0.80 |
| **Peer distribution** | 0.84 | 0.14 | 0.01 | 0.00 |
| **Partner distribution** | 0.15 | 0.80 | 0.04 | 0.00 |
| **Peer educator distribution** | 0.01 | 0.05 | 0.74 | 0.20 |

## S4 Table: Linkage to ART or Any Care Among people living with HIV by Distribution Strategy, Study Design and Population Subgroup

| **Strategy** | **Design** | **Population Type** | **Pooled Risk Ratio** | **Studies** |
| --- | --- | --- | --- | --- |
| Peer distribution | Cohort | MSM | 0.99 [0.78, 1.27] | Van Der Elst 2017 |
| Partner distribution | RCT | Male partners of ANC clients | 0.69 [0.38, 1.27] | Choko 2019, Choko 2021 a, Masters 2016, Korte 2020 |
|  | RCT | Partners of people living with HIV | 0.79 [0.60, 1.04] | Joseph 2022, Mujugira 2022, Dovel 2019 |
| Peer educator distribution | RCT | FSW | 0.80 [0.63, 1.02] | Chanda 2017, Ortblad 2017 |

Abbreviations: ANC, antenatal care clinic; FSW, female sex workers; HIV, human immunodeficiency virus; MSM, men who have sex with men; RCT, randomized controlled trial.

## S5 Table: Risk of bias for included studies - Observational studies

| Study | Representativeness of exposed cohort | Selection of non-exposed cohort | Ascertainment of exposure | Demonstration that outcome of interest was not present at start of study | Cohorts comparable | Assessment of outcome | Length of follow-up | Loss to follow-up rate | Overall Quality |
| --- | --- | --- | --- | --- | --- | --- | --- | --- | --- |
| Kwan 2023 | * | - | * | * | - | * | * | * | Good |
| Kitenge 2022 | * | * | * | * | - | * | * | - | Good |
| Lightfoot 2018 | * | - | * | - | * | * | - | * | Fair |
| Lippman 2018 | * | * | * | * | * | - | * | - | Good |
| Li S 2021 | * | - | * | * | - | - | * | - | Poor |
| Matovu 2020 | * | - | * | * | - | - | * | * | Fair |
| Nasuuna 2022 | * | - | * | * | - | - | * | * | Fair |
| Nguyen 2019 1 | * | * | - | - | - | * | - | - | Poor |
| Nguyen 2019 2 | * | * | * | - | - | * | - | - | Poor |
| Okoboi 2020 | * | * | * | * | - | * | * | - | Good |
| Pintye 2019 | * | - | * | * | - | - | * | - | Poor |
| Thirumurthy 2016 | * | * | * | * | - | * | * | * | Good |
| Van Der Elst 2017 | * | * | * | - | - | - | * | * | Fair |
| Wu D 2021 | * | - | * | * | - | * | * | * | Good |
| Zhang J 2021 | * | - | * | * | - | - | * | * | Fair |
| Zishiri 2022 | * | - | * | * | - | - | * | * | Fair |

## S6 Table: Risk of bias for included studies – RCTs and a quasi-experimental study

| Study | Random sequence generation (selection bias) | Allocation concealment (selection bias) | Blinding of participants and personnel (performance bias) | Blinding of outcome assessment (detection bias) | Incomplete outcome data (attrition bias) | Selective reporting (reporting bias) | Other bias |
| --- | --- | --- | --- | --- | --- | --- | --- |
| Chanda 2017 | Low | Low | Low | Low | Low | Low | Low |
| Choko 2019 | Low | Low | High | High | Low | Low | Low |
| Choko 2021 | Low | Low | High | Low | Low | Low | Low |
| Dovel 2019 | Unclear | Unclear | High | High | Low | Low | Unclear |
| Frye 2021 | Low | Low | High | High | Low | Low | Low |
| Gichangi 2018 | Low | Low | High | High | Low | Low | Low |
| Joseph 2022 | Low | Low | High | Unclear | Low | Low | Low |
| Korte 2020 | Low | Low | High | High | Low | Low | Low |
| MacGowan 2020 | Low | Low | High | High | High | Low | Unclear |
| Marwa 2019 | Low | Low | High | High | Low | Low | Low |
| Masters 2016 | Low | Low | High | High | Low | Low | Low |
| Merchant 2018 | Low | Unclear | High | High | Unclear | Low | Low |
| Mujugira 2022 | Low | Unclear | High | High | Unclear | Low | Unclear |
| Ortblad 2017 | Low | Low | Low | Low | Low | Low | Low |
| Pettifor 2020 | Low | Low | High | High | Low | Low | Low |
| Sha 2022 | High | High | High | Unclear | High | Low | Low |
| Shahmanesh 2021 | Low | Low | High | Low | Unclear | Low | Low |
| Thirumurthy 2021 | Low | Unclear | High | High | Low | Low | Low |
| Wango 2023 | Low | Low | High | Unclear | Low | Low | Low |
| Young 2022 | Low | Low | High | Unclear | Low | Low | Low |
| Young 2013 | Low | Unclear | High | High | Unclear | Low | Low |
| Zhang 2020 | Low | Low | Low | Unclear | Low | Low | Low |
| Zhou 2022 | Low | Low | High | Low | Low | Low | Low |

## S1 Figure: Risk of bias for included studies – RCTs and a quasi-experimental study


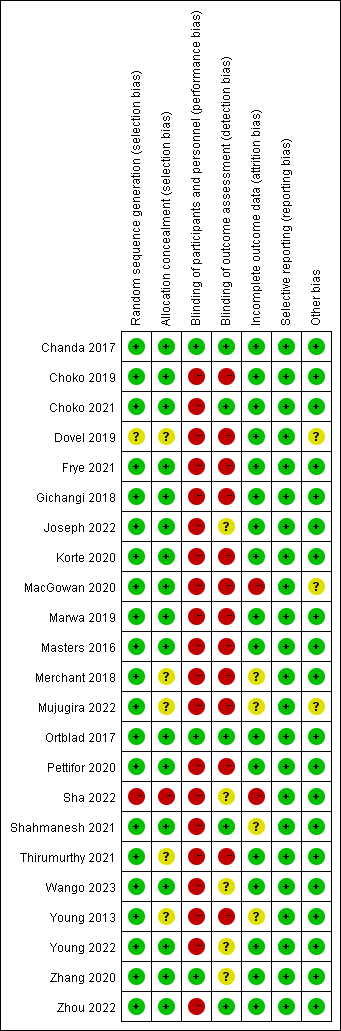


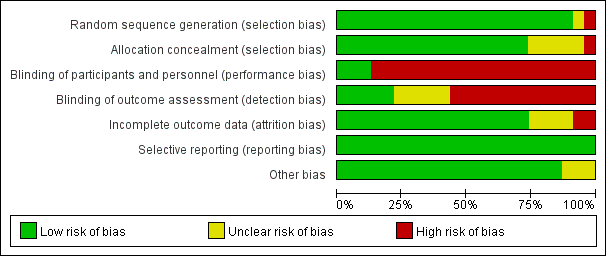


Figure S1. Cochrane risk of bias quality assessment for included studies: RCTs
